# Estimation of the adjusted cause‐specific cumulative probability using flexible regression models for the cause‐specific hazards

**DOI:** 10.1002/sim.8209

**Published:** 2019-06-18

**Authors:** Dimitra‐Kleio Kipourou, Hadrien Charvat, Bernard Rachet, Aurélien Belot

**Affiliations:** ^1^ Cancer Research UK Cancer Survival Group, Faculty of Epidemiology and Population Health, Department of Non‐Communicable Disease Epidemiology London School of Hygiene and Tropical Medicine London UK; ^2^ Division of Prevention, Center for Public Health Sciences National Cancer Center Tokyo Japan

**Keywords:** cause‐specific hazards, competing risks, cumulative incidence function, cumulative probability of death, flexible parametric models

## Abstract

In competing risks setting, we account for death according to a specific cause and the quantities of interest are usually the cause‐specific hazards (CSHs) and the cause‐specific cumulative probabilities. A cause‐specific cumulative probability can be obtained with a combination of the CSHs or via the subdistribution hazard. Here, we modeled the CSH with flexible hazard‐based regression models using B‐splines for the baseline hazard and time‐dependent (TD) effects. We derived the variance of the cause‐specific cumulative probabilities at the population level using the multivariate delta method and showed how we could easily quantify the impact of a covariate on the cumulative probability scale using covariate‐adjusted cause‐specific cumulative probabilities and their difference. We conducted a simulation study to evaluate the performance of this approach in its ability to estimate the cumulative probabilities using different functions for the cause‐specific log baseline hazard and with or without a TD effect. In the scenario with TD effect, we tested both well‐specified and misspecified models. We showed that the flexible regression models perform nearly as well as the nonparametric method, if we allow enough flexibility for the baseline hazards. Moreover, neglecting the TD effect hardly affects the cumulative probabilities estimates of the whole population but impacts them in the various subgroups. We illustrated our approach using data from people diagnosed with monoclonal gammopathy of undetermined significance and provided the R‐code to derive those quantities, as an extension of the R‐package mexhaz.

## 1 INTRODUCTION

In survival analysis, the one‐to‐one relationship between the risk of an event (probability scale) and the rate at which the event occurs (hazard scale) is well known when studying a single event/cause. This is a key feature in hazard regression models in order to examine how covariates affect the survival probability.^1^ Thus, assuming that the survival time *t* of an individual can be described by a positive random variable *T* with probability density function *f*, the cumulative distribution function *F* is defined as $F(t)=P(T\leq t)=\int_{0}^{t}f(u)\text{du}=\int_{0}^{t}\lambda (u)S(u)\text{du}$, where *S*(*t*)=*P*(*T*>*t*)=1−*F*(*t*) is the survival probability and *λ*(*t*) is the hazard function.

However, in the competing risks setting where more than one cause are acting, the (total) hazard is the sum of all cause‐specific hazards (CSHs) $\lambda (t)=\sum_{j=1}^{J}\lambda_{j}(t)$, where the cause *j*‐specific hazard *λ*_*j*_ represents the *rate of failure from cause j per time unit for individuals who are still at risk*.^2^, ^3^ The cumulative probability of dying from a particular cause until time *t* in the presence of all other causes (also called the cumulative incidence function) depends on all the CSHs

$$F_{j}(t)=P(T\leq t,\text{Cause}=j)=\overset{t}{\underset{0}{\int }}\lambda_{j}(u)S(u)\text{du},$$

where *S* is the overall survival (ie, from all causes), $S(t)=\exp\left(-\int_{0}^{t}\sum_{j=1}^{J}\lambda_{j}(u)du\right)$. As a result, the one‐to‐one relationship between the rate and the risk for a given cause is now lost, since in this case, the risk of a given cause is affected by all CSHs. Therefore, a covariate effect in a CSH model cannot be directly translated to an effect on the cause‐specific cumulative probability.^4^, ^5^

Nevertheless, the cause‐specific cumulative probability is of great interest in competing risks case since it quantifies the cumulative probability (risk) in the presence of other causes,^6^, ^7^ thus being a useful overall measure of prognosis for the patients.^8^ Cause‐specific cumulative probabilities can be estimated nonparametrically using the Aalen‐Johansen estimator^3^, ^7^ or the so‐called subdistribution hazard with the Fine and Gray model.^3^, ^9^ However, our focus here is on its estimation via CSH regression models as this approach allows the estimation of both CSHs (rate) and cause‐specific cumulative probabilities (risk). CSH regression models are quite useful because they are easy to fit (since we only need to censor for the competing event),^4^ and unlike models on subdistribution hazard, they give a simple interpretation of parameter estimates: CSH ratios measure the impact of the risk factors on the rate that correspond to a given cause of death.^10^ Moreover, there is a wide variety of models that we can apply, which range from the simple Cox proportional hazards models^11^ to the more sophisticated flexible regression models including time‐dependent (TD) effects.^12^ Lastly, another advantage of modeling on the CSH scale is the possibility to estimate easily the covariate‐adjusted cause‐specific cumulative probabilities,^13^, ^14^ which can be used to further calculate standardized risk differences.

In this paper, we focus on the use of flexible regression models for the CSH. Section 2 details how to obtain individual‐ and population‐level smooth estimates of the cause‐specific cumulative probabilities, along with their variances, using the parameter estimates from CSH models. We also explain how this approach can be easily employed for deriving directly adjusted cause‐specific cumulative probabilities. In Section 3, we provide results from a simulation study assessing the performance of the approach in its ability to estimate the cause‐specific cumulative probabilities, depending on whether the proportional hazard assumption for one CSH is correct or not. In Section 4, we provide an illustrative example using data from individuals diagnosed with monoclonal gammopathy of undetermined significance (MGUS) that are provided in the mgus2 dataset from the R‐package survival. Finally, we discuss the results and present ideas for further research.

## 2 METHODS

### 2.1 Flexible hazard‐based regression model

#### 2.1.1 Cause‐specific hazard model

The regression model used for the CSH is defined on the log‐hazard scale. It was first described by Remontet et al^15^ and recently extended by Charvat et al.^16^ In its general formulation, this model uses B‐spline functions for modeling the logarithm of the baseline hazard parameters ***γ***_*j*_ and the time‐dependent (cause *j*)‐specific hazard ratios ***α***_*j*_(*t*) for the corresponding vector of covariates **x**. Thus, the model for the (logarithm of) the (cause *j*)‐specific hazard may be written as

$$\log[\lambda_{j}(t,\text{x};\beta_{j})]=\log(\lambda_{0}(t;\gamma_{j}))+{\text{x}}^{⊤}\alpha_{j}(t),$$

where ***β***_*j*_ is a vector of parameters, which includes the parameters for (i) the baseline hazard and (ii) the time‐dependent (cause *j*)‐specific hazard ratios, ie, $\beta_{j}={\left(\gamma_{j}^{⊤},\alpha_{j}^{⊤}\right)}^{⊤}$. We advise the reader to refer to section 2.1 in the work of Charvat et al^16^ and the references mentioned therein for more details on splines and knots selection.

#### 2.1.2 Likelihood function

For an individual *i*, denote by *t*_*i*_ the observed follow‐up time, *δ*_*i*_ the failure indicator (0 for censoring and 1 for death), *j*_*i*_ the type of event among *J* different types (set *j*_*i*_=0 for censored times), and **x**_*i*_ a vector of covariates. Therefore, our sample may be defined as $\left\{t_{i},\delta_{i},j_{i},{\text{x}}_{i}\right\}$, *i*=1,…,*N*.

In a competing risks setting with *J* different causes, and assuming a random censoring mechanism, the likelihood function may be written as^2^, ^17^

(1) $$\overset{N}{\underset{i=1}{\prod }}{\left[\lambda_{j_{i}}(t_{i},{\text{x}}_{i})\right]}^{\delta_{i}}S(t_{i},{\text{x}}_{i})=\overset{J}{\underset{j=1}{\prod }}\overset{N}{\underset{i=1}{\prod }}{\left[\lambda_{j}(t_{i},{\text{x}}_{i})\right]}^{\delta_{ij}}\exp\left(-\overset{t_{i}}{\underset{0}{\int }}\lambda_{j}(u,{\text{x}}_{i})\text{du}\right),$$

where *δ*_*ij*_ is equal to 1 if cause *j* was observed to happen at time *t*_*i*_, and 0 otherwise.

The likelihood can be factorized as the product of *J* cause‐specific likelihood as defined in the right‐hand side of formula 1. Therefore, one can fit a hazard‐based regression model for cause *j* on the observed data for statistical inference, treating all failure times other than *j* as censored without relying on any assumption of independent competing risks.^1^, ^3^ By doing so, the contribution to the log‐likelihood for individual *i* when estimating the (cause *j*)‐specific hazard is

(2) $$l_{i}^{j}(\beta_{j})=-\overset{t_{i}}{\underset{0}{\int }}\lambda_{j}(u,{\text{x}}_{i};\beta_{j})\text{du}+\delta_{ij}\log\left[\lambda_{j}(t_{i},{\text{x}}_{i};\beta_{j})\right].$$

### 2.2 Estimation of the cumulative probabilities of death from each cause

We show in the following how we estimated the cumulative probability of death for each cause, after plugging‐in the estimated parameters of the CSH models. We start with the estimation for a given set of observed covariates (individual‐level predictions) and then move on to the estimation of the cumulative probabilities of death from each cause in the whole population. To simplify the notation without loss of generality, we consider only two different causes, denoted as *j* and $\bar{j}$.

#### 2.2.1 Individual‐level prediction of the probability of death from a given cause

##### 2.2.1.1 Point estimates

To estimate *F*_*j*_ at time *t* for an individual *i* with an observed vector of covariates **x**_*i*_, the CSHs need to be combined. We use the (cause *j*)‐specific vectors of parameters ${\hat{\beta }}_{j}$ of length *p*_*j*_ and define the total vector of parameters, $\hat{\beta }={\left({\hat{\beta }}_{j}^{⊤},{\hat{\beta }}_{\bar{j}}^{⊤}\right)}^{⊤}$ of length $m=p_{j}+p_{\bar{j}}$.

(3) $$\begin{array}{ccc} {\hat{F}}_{j}\left(t,{\text{x}}_{i};\hat{\beta }\right) & =\overset{t}{\underset{0}{\int }}S\left(u,{\text{x}}_{i};\hat{\beta }\right)\lambda_{j}\left(u,{\text{x}}_{i};{\hat{\beta }}_{j}\right)\mathrm{du} & \\ & =\overset{t}{\underset{0}{\int }}\exp\left(-\overset{u}{\underset{0}{\int }}\lambda_{j}\left(\nu ,{\text{x}}_{i};{\hat{\beta }}_{j}\right)+\lambda_{\bar{j}}\left(\nu ,{\text{x}}_{i};{\hat{\beta }}_{\bar{j}}\right)d\nu \right)\lambda_{j}\left(u,{\text{x}}_{i};{\hat{\beta }}_{j}\right)\mathrm{du}\\ & =\overset{t}{\underset{0}{\int }}S_{j}\left(\nu ,{\text{x}}_{i};{\hat{\beta }}_{j}\right)S_{\bar{j}}\left(\nu ,{\text{x}}_{i};{\hat{\beta }}_{\bar{j}}\right)\lambda_{j}\left(u,{\text{x}}_{i};{\hat{\beta }}_{j}\right)\mathrm{du} \end{array}$$

##### Variance

The variance of the cause‐specific cumulative probabilities is derived via the multivariate delta method. The delta method is a general approach that allows to approximate the variance of a differentiable function *ϕ* of the estimated parameters as

$$\mathrm{Var}\left[\phi \left(\hat{\beta }\right)\right]\approx {\left[{\nabla \phi (\beta )}_{|\beta =\hat{\beta }}\right]}^{⊤}\Sigma_{\beta }\left[\nabla \phi {\left(\beta \right)}_{|\beta =\hat{\beta }}\right],$$

where **Σ**_***β***_ is the parameters covariance matrix and *∇* *ϕ* is the gradient of *ϕ*, ie, the vector of first derivatives of *ϕ*.

$${\nabla \phi (\beta )}_{|\beta =\hat{\beta }}={\left(\frac{\partial \phi }{\partial \beta_{1}}\left(\hat{\beta }\right),\text{…}\;,\frac{\partial \phi }{\partial \beta_{p}}\left(\hat{\beta }\right)\right)}^{⊤}$$

So, in our case, the variance of *F*_*j*_ at time *t* for the vector of covariables **x** and based on the vector of estimated parameters $\hat{\beta }$ would be approximated by

$$\mathrm{Var}\left[F_{j}\left(t,\text{x};\hat{\beta }\right)\right]={\left[\nabla F_{j}{\left(t,\text{x};\hat{\beta }\right)}_{|\beta =\hat{\beta }}\right]}^{⊤}{\hat{\Sigma }}_{\beta }\left[\nabla F_{j}{\left(t,\text{x};\hat{\beta }\right)}_{|\beta =\hat{\beta }}\right].$$

Details on how to derive this quantity can be found in Appendix 1.B.

It is possible to obtain the approximate 100∗(1−*α*)% confidence interval of 1−*F*_*j*_(*t*,**x**;***β***) based on the (approximate) normality assumption as

(4) $$\mathrm{Var}\left[\log\left(-\log\left(1-F_{j}(t,\text{x};\beta )\right)\right)\right]=\frac{\mathrm{Var}\left[1-F_{j}(t,\text{x};\beta )\right]}{{\left(\log\left(1-F_{j}(t,\text{x};\beta )\right)\left(1-F_{j}(t,\text{x};\beta )\right)\right)}^{2}},$$

where Var[1−*F*_*j*_(*t*,**x**;***β***)]=Var[*F*_*j*_(*t*,**x**;***β***)]. We opted to estimate the approximate confidence intervals based on the complementary of *F*_*j*_ in order to avoid problems in the denominator due to small probabilities coming as a result of rare events or for probability inference shortly after diagnosis.

After backtransforming, the approximate 100∗(1−*α*)% confidence intervals can be estimated as

(5) $$F_{j}{\left(t,\text{x};\beta \right)}^{\Omega },$$

where $\Omega =\{\exp\{\pm z_{\alpha }\mathrm{Var}[\log(-\log(F_{j}(t,\text{x};\beta )))]\}\}$ and *z*_*α*_ is the (1‐*α*/2) quantile of the standard normal distribution.

#### 2.2.2 Population‐level prediction of the probability of death from a given cause

##### Point estimates

To obtain the population value of the cumulative incidence function for cause *j* at time *t*, we need to compute the average of the *N* individual predicted cumulative probabilities of death from cause *j*.

$${\hat{F}}_{j}^{P}(t;\beta )=\frac{1}{N}\overset{N}{\underset{i=1}{\sum }}{\hat{F}}_{j}(t,{\text{x}}_{i};\beta )$$

##### Variance

The key point here is to account for the correlation of the *N* individual‐predicted cumulative probabilities because they were obtained from the same vector of estimated parameters $\hat{\beta }$.^18^, ^19^ By applying the multivariate delta method, we obtain a variance estimation of the population value

$$\mathrm{Var}\left[F_{j}^{P}(t;\beta )\right]={\text{w}}^{⊤}{\left[\nabla F_{j}^{\text{Mat}}{\left(t;\beta \right)}_{|\beta =\hat{\beta }}\right]}^{⊤}{\hat{\Sigma }}_{\beta }\left[\nabla F_{j}^{\text{Mat}}{\left(t;\beta \right)}_{|\beta =\hat{\beta }}\right]\text{w},$$

where **w** is a column vector of *N* weights (in our case, all equal to 1/*N*), and $\nabla F_{j}^{\text{Mat}}(t,\beta )$ is a (*m*×*N*) matrix

$$\nabla F_{j}^{\text{Mat}}(t;\beta )=\left(\nabla F_{j}{\left(t,{\text{x}}_{1};\beta \right)}_{|\beta =\hat{\beta }},\text{…}\;,\nabla F_{j}(t,{{\text{x}}_{N};\beta )}_{|\beta =\hat{\beta }}\right),$$

with its elements defined in formula 4 of Appendix 1.B. The 100∗(1−*α*)% confidence interval is obtained using formulae 4 and 5.

#### 2.2.3 Adjusted cumulative probability of death from cause j and their difference

Using our approach, we can extend the idea of adjusted survival curve^14^, ^19^^‐^ 22 to the competing risks setting for providing the adjusted cumulative probability of death estimates from a given cause $F_{j}^{\text{Adj}}$.^23^ It would provide a quantity that is directly standardized to the empirical distribution of the covariates observed in the study^24^ and may be further used to calculate the standardized risk difference.^25^ $F_{j}^{\text{Adj}}$ is useful when there is an effect of interest, eg, treatment, and the treatment groups are imbalanced with respect to factors influencing the CSH.^14^ Another advantage is that we can quantify the effect of a specific covariate of interest on the probability scale (cumulative incidence). Although the covariate effect on the CSH is not directly linked to the probability scale, with the adjusted probabilities it is possible to translate the covariate effect on the probability scale by quantifying both the “direct” effect of a variable on the CSH of interest, as well as the “indirect” effect of this variable on the competing hazard.^26^

To compute the $F_{j}^{\text{Adj}}$, we predict the *F*_*j*_ for each individual using their observed covariates (except for the variable we are interested in, which is set to a specific value), and then, we average the estimates.^19^ For example, if the use of a specific drug (say, A or B) is the exposure of interest, then we can construct hypothetical populations where all individuals keep their characteristics **z** (**z**⊂**x**) as observed except that the drug is set to drug *K*={*A*,*B*}.

$$F_{j}^{\text{Adj}}(t,\text{Drug}=K,\text{z})=\frac{1}{N}\overset{N}{\underset{i=1}{\sum }}{\hat{F}}_{j}(t,\text{Drug}=K,{\text{z}}_{i})$$

By taking the difference between $F_{j}^{\text{Adj}}(t,\text{Drug}=A,\text{z})$ and $F_{j}^{\text{Adj}}(t,\text{Drug}=B,\text{z})$, we can quantify the average treatment effect on probability scale as

(6) $$D(t;\beta )=E\left[F_{j}^{\text{Adj}}(t,A,z;\beta )-F_{j}^{\text{Adj}}(t,B,z;\beta )\right]=E\left[F_{j}^{\text{Adj}}(t,A,z;\beta )\right]-E\left[F_{j}^{\text{Adj}}(t,B,z;\beta )\right].$$

The 100∗(1−*α*)% confidence interval is obtained as $\hat{D}(t)\;\pm \;z_{\alpha }\sqrt{\text{Var}(\hat{D}(t))}$, where

$$\text{Var}[D(t;\beta )]={\text{w}}^{⊤}{\left[\nabla D{\left(t;\beta \right)}_{|\beta =\hat{\beta }}^{\text{Mat}}\right]}^{⊤}{\hat{\Sigma }}_{\beta }\left[\Delta D{\left(t;\beta \right)}_{|\beta =\hat{\beta }}^{\text{Mat}}\right]\text{w}.$$

For more information on the variance calculation, the reader is referred to Appendix 1.C.

The same procedure can be repeated for the other cause $\bar{j}$, and more generally, we can get as many estimands as the number of competing events in the data.^26^ We can also apply this direct standardization process to a subsample of the entire population (eg, the treated) if the interest is on standardizing only on the treated individuals or even use an external (reference) population.

### 2.3 Implementation

To implement those theoretical quantities, we made some choices presented in the following.

Firstly, the log‐likelihood (formula 2) cannot be evaluated analytically because the integral defining the cumulative hazard, $\int_{0}^{t_{i}}\lambda_{j}(u,\text{x})\text{du}$, does not have a closed analytical form. In our approach, we used the Gauss‐Legendre (G‐L) quadrature, which is a numerical integration technique that approximates the integral of a function defined in [−1,1] with a weighted sum using *K* pre‐specified weights and nodes. By applying a simple change of variable, we can approximate the integral of a function *g* on any bounded domain [*a*,*b*] on which it is defined by using the following formula:

$$\overset{b}{\underset{a}{\int }}g(t,\text{x})dt\approx \frac{b-a}{2}\overset{K}{\underset{k=1}{\sum }}w_{k}^{K}g\left(\frac{a+b}{2}+\frac{b-a}{2}z_{k}^{K},\text{x}\right),$$

where $w_{k}^{K}$ and $z_{k}^{K}$ are the weights and abscissas for the *K*‐point G‐L rule, respectively. We used the G‐L quadrature to calculate the cumulative hazards in formula 2, and the maximum likelihood estimates were obtained using a Newton‐type algorithm for the optimization (function nlm in R).

Because we estimated the parameters separately for each cause, we ended up with a *m*×*m* block diagonal covariance matrix **Σ**_***β***_ with two blocks ${\hat{\Sigma }}_{\beta_{j}}$ and ${\hat{\Sigma }}_{\beta_{\bar{j}}}$ corresponding to the covariance matrix returned from each CSH model.

The cause‐specific flexible hazard regression models were fitted using the R‐package mexhaz with some additional R‐functions programmed in R. The R‐code to implement our approach for the data used in the illustrative example is provided in Appendix 2.

## 3 SIMULATION STUDY

We performed a simulation study to evaluate the frequentist properties of our approach based on flexible regression models for the CSH in its ability to estimate the cumulative probabilities of death from each cause. To quantify the impact of neglecting a TD effect on the estimation of the cause‐specific cumulative probabilities, we simulated two scenarios: scenario 1 where the proportional hazards assumption is met and scenario 2 where a TD effect was simulated on the cancer‐specific hazard. We also evaluated the coverage properties of the detailed confidence intervals using the multivariate delta method for the cumulative probabilities at population level.

### 3.1 Data generation and simulation design

For each scenario, we simulated *n*_sim_=500 datasets with sample size of *N*={300,1000}. Each individual was assigned a vector of three covariates that included information about sex, year of diagnosis, and age at diagnosis. Sex was simulated as a binary covariate drawn from a Bernoulli distribution with probability 0.5 in scenario 1 and 0.3 (of being a woman) in scenario 2. This choice was made based on the fact that a TD effect of sex was simulated in scenario 2 and an unbalanced distribution of sex would be more appropriate in order to test the performance of the method. Year of diagnosis was simulated as a continuous variable and sampled from a uniform distribution, ranging from 2000 to 2003. Age was simulated as a continuous variable by first selecting an age class according to predefined probabilities (0.25 for age class [30, 65), 0.35 for age class [65, 75), and 0.40 for age class [75, 80)) and then sampling from a class‐specific uniform distribution.^17^

The two scenarios tried to mimic typical real situations for colon cancer patients. We generated two independent simulating processes as to account for two causes, namely, death from colon cancer and death from other causes.

We chose a Generalized Weibull distribution with parameters (*κ*,*ρ*,*α*) for the cancer‐specific survival time (*T*_*C*_), and we used the inverse probability transform method.^17^, ^27^ For individual *i*, the cancer‐specific hazard used to simulate *T*_*C*_ in scenario 1 was defined as $\lambda_{C}(t,{\text{Age}}_{i},{\text{Sex}}_{i})=\lambda_{0}(t)\exp\left\{\beta_{\text{Age}}{\text{Age}}_{i}+\beta_{\text{Sex}}{\text{Sex}}_{i}\right\}$ where $\lambda_{0}(t)=\frac{\kappa \rho^{\kappa }t^{\kappa -1}}{1+\frac{{\left(\rho t\right)}^{\kappa }}{\alpha }}$. In scenario 1, the parameters (*κ*,*ρ*,*α*) for the baseline hazard were equal to (2, 1.2, 0.1), and the values used for the covariate parameters were *β*_Age_=0.03 (for 1 year increase) and *β*_Sex_=0.3. In scenario 2, we used a sex‐specific baseline hazard (which leads to a TD effect of sex), with the simulated parameters (*κ*,*ρ*,*α*) set to (2, 0.4, 0.2) for men and (2, 0.3, 0.2) for women, with *β*_Age_=0.03 (for 1 year increase) for both sexes.

The time to death from other causes ( $T_{\bar{C}}$) was simulated assuming a piecewise exponential distribution with the rates coming from the population mortality rates obtained from the UK age‐ and sex‐specific life tables. We set the administrative censoring time (*C*) at 10 years and a separate distribution for the dropouts following an exponential distribution (*λ*_*d*_=0.035) as to account for approximately 15% of people lost to follow‐up, while the total amount of censoring in each dataset was on average around 38%. The final survival time (*T*) was obtained as $T=\min(T_{C},T_{\bar{C}},C)$. A vital status indicator *δ* was created, *δ*=0 for individual censored at *T*, and *δ*=1 for those being dead at time *T* (whatever the cause). Additionally, the cause of death *j* was denoted as *j*=1 for death from cancer and *j*=2 for death from other causes.

The true values ${\tilde{F}}_{C}(t)$ and ${\tilde{F}}_{\bar{C}}(t)$ of the cumulative probabilities of death from cancer and from other causes were obtained at time *t*=1,5,10 years, calculated using the G‐L quadrature. Although we assumed the same covariate distribution in both simulations (*N* = 300 and *N* = 1000), the true values differ slightly because they rely on different simulated individuals.

### 3.2 Analysis of simulated data

The simulated data were analyzed with a nonparametric method and with the flexible regression models for the log of the baseline hazard. In scenario 1, we tested two flexible regression models for the logarithm of the cancer‐specific hazard: (a) a model with a quadratic B‐spline baseline hazard function and knots at 1 and 5 years and (b) a model with cubic B‐spline baseline hazard function with same knots. The explanatory variables in both models were age at diagnosis and sex. We omitted the year of diagnosis since we did not simulate an effect on the cancer‐specific hazard (its range was very small and it was mainly used to retrieve the population mortality rates from the UK life tables). In scenario 2, we used the same models as in scenario 1 (models (a) and (b)) but also an additional model (model (c)) that had a cubic B‐spline for the baseline hazard function (with two knots at 1 and 5 years) and a TD effect for sex, which was modeled also with a cubic B‐spline with two knots at 1 and 5 years. The knots were located at these points based on our previous experience analyzing cancer survival data.^28^, ^29^ The model for the hazard of other causes was kept in all cases the same, with a baseline hazard modeled with a quadratic B‐spline with one knot at 1 year.

We assessed the performance of the aforementioned methods in their ability to estimate the probabilities of death from cancer and death from other causes at *t*={1,5,10} years after diagnosis. We calculated the following quantities: (i) the bias, defined as the difference between the average of the *n*_sim_=500 estimated values and the true value $\tilde{\theta }$: $\frac{1}{n_{\text{sim}}}\sum_{i=1}^{n_{\text{sim}}}{\hat{\theta }}_{i}-\tilde{\theta }$; (ii) the relative bias, expressed as the bias divided by the true value and multiplied by 100; (iii) the empirical standard error $\sqrt{\frac{1}{n_{\text{sim}}-1}\sum_{i=1}^{n_{\text{sim}}}{\left({\hat{\theta }}_{i}-\bar{\theta }\right)}^{2}}$ where $\bar{\theta }=\sum_{i=1}^{n_{\text{sim}}}{\hat{\theta }}_{i}$; (iv) the model standard error $\sqrt{\frac{1}{n_{\text{sim}}}\sum_{i=1}^{n_{\text{sim}}}\hat{\text{Var}}(\hat{\theta_{i}})}$; (v) the root mean squared error (RMSE) $\sqrt{\frac{1}{n_{\text{sim}}}\sum_{i=1}^{n_{\text{sim}}}{\left({\hat{\theta }}_{i}-\tilde{\theta }\right)}^{2}}$; and (vi) the coverage probability that is the proportion of samples in which the 95% confidence interval included $\tilde{\theta }$.

Our computations were performed in R 3.2.0. We used the nonparametric method for the cumulative probability provided by R‐package cmprsk (version 3.4.2, function cuminc), while flexible parametric models were estimated using R‐package mexhaz (version 1.5, function mexhaz).

### 3.3 Results

#### Performance on the whole population

Although sample size did not seem to affect the general performance of the method in scenario 1, density plots (see Figure A1.4) showed that the models that were applied to bigger sample size datasets resulted in more accurate estimates as expected. In all methods, the relative bias and the RMSEs were very low, the model standard errors (ModSE) were quite close to the empirical ones (empSE), and the majority of the coverage probabilities were within the acceptable coverage probability range ([0.931, 0.969])^30^ (see Table 1). Exception to that is model (a) at the 1st year for the 1st cause. This could be explained by the fact that the model with a quadratic B‐spline was not flexible enough and the estimated baseline hazard could not approximate adequately the sudden peak of the simulated baseline hazard during the 1st year (see Figure A1.1).

**Table 1:** Simulation results for the population cause‐specific cumulative probabilities based on 500 simulated datasets with sample size of N={300,1000} for scenario 1. The performance measures are given for the nonparametric method (obtained via R‐package cmprsk) and for the flexible hazard‐based regression models (model (a) and model (b)). Model (a) has a quadratic B‐spline baseline hazard function and knots at 1 and 5 years, whereas model (b) has a cubic B‐spline baseline hazard function with the same knots. The explanatory variables in both models were age and sex

**Method**	**Cause**	**Time**	**True Value**		**Relative**	**Bias(%)**	**empSE**		**RMSE**		**ModSE**		**Coverage** ^*†*^	
			*N* = 300	*N* = 1000	*N* = 300	*N* = 1000	*N* = 300	*N* = 1000	*N* = 300	*N* = 1000	*N* = 300	*N* = 1000	*N* = 300	*N* = 1000
		1	0.2681	0.2637	0.1868	−0.0127	0.0264	0.0144	0.0258	0.0141	0.0264	0.0144	0.950	0.942
	1	5	0.4672	0.4604	−0.1607	−0.2148	0.0292	0.0159	0.0297	0.0162	0.0292	0.0160	0.954	0.950
Nonparametric		10	0.5209	0.5139	−0.1417	−0.2639	0.0293	0.0157	0.0303	0.0166	0.0293	0.0158	0.952	0.966
		1	0.0249	0.0243	1.1910	0.7154	0.0090	0.0049	0.0091	0.0049	0.0090	0.0049	0.966	0.952
	2	5	0.0925	0.0903	−0.2632	0.8874	0.0166	0.0093	0.0174	0.0095	0.0166	0.0093	0.962	0.944
		10	0.1636	0.1600	−0.3291	0.8568	0.0208	0.0123	0.0232	0.0126	0.0208	0.0124	0.974	0.942
		1	0.2681	0.2637	−2.1076	−2.6419	0.0246	0.0135	0.0233	0.0127	0.0252	0.0152	0.944	0.909
	1	5	0.4672	0.4604	−0.8702	−0.9620	0.0287	0.0155	0.0284	0.0155	0.0289	0.0161	0.944	0.950
Model (a)		10	0.5209	0.5139	−0.3591	−0.4644	0.0293	0.0156	0.0295	0.0161	0.0294	0.0157	0.946	0.960
		1	0.0249	0.0243	0.3985	0.7352	0.0078	0.0042	0.0078	0.0042	0.0078	0.0042	0.946	0.948
	2	5	0.0925	0.0903	−0.1481	0.3033	0.0153	0.0086	0.0159	0.0087	0.0153	0.0087	0.954	0.960
		10	0.1636	0.1600	−0.6713	0.4795	0.0206	0.0121	0.0222	0.0121	0.0206	0.0121	0.960	0.940
		1	0.2681	0.2637	0.9643	0.5645	0.0257	0.0141	0.0243	0.0132	0.0258	0.0142	0.938	0.936
	1	5	0.4672	0.4604	−0.5055	−0.5885	0.0289	0.0156	0.0284	0.0155	0.0290	0.0158	0.948	0.946
Model (b)		10	0.5209	0.5139	−0.3172	−0.4245	0.0293	0.0156	0.0295	0.0162	0.0294	0.0157	0.946	0.960
		1	0.0249	0.0243	0.1221	0.4446	0.0078	0.0042	0.0078	0.0042	0.0078	0.0042	0.946	0.950
	2	5	0.0925	0.0903	0.0670	0.5442	0.0152	0.0087	0.0160	0.0087	0.0152	0.0087	0.954	0.956
		10	0.1636	0.1600	−0.6301	0.5274	0.0206	0.0121	0.0222	0.0121	0.0206	0.0121	0.960	0.940

Abbreviations: empSE, empirical standard error; ModSE, model standard error; RMSE, root mean square error.

*†* Acceptable coverage range is [0.931,0.969] (calculated based on the work of Burton et al^30^)

**Figure 1:**
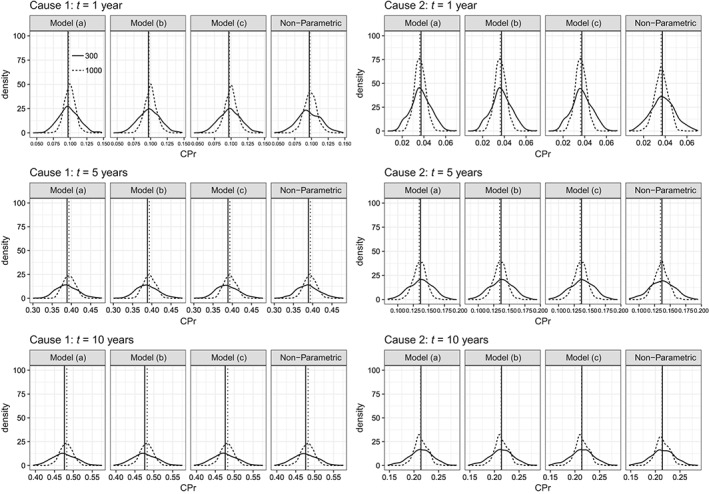
Empirical distribution of the 500 parameter estimates of cumulative probabilities for each model and each cause at 3 timepoints: 1, 5, and 10 years in scenario 2. Vertical lines denote the true values. Model (a) has a quadratic B‐spline baseline function and knots at 1 and 5 years, whereas model (b) has a cubic B‐spline baseline function with the same knots. The explanatory variables in FPM models were age and sex. Models (b) and (c) have the same baseline function (cubic B‐spline with knots at 1 and 5 years), but model (b) has a fixed effect for sex, whereas model (c) has a TD effect for sex, which is modeled with a cubic B‐spline with two knots at 1 and 5 years

In scenario 2, where a TD effect of sex was simulated, models (a) and (b) were misspecified, while model (c) was properly specified. Interestingly, all methods performed well in terms of overall performance on the whole population. In all cases, relative bias was low, empSE was very close to the ModSE, and RMSEs were all quite similar for a given time (see Table 2). Estimates were nicely spread around the true values regardless of the sample size used, while higher accuracy was achieved when sample size was equal to 1000 (see Figure 1).

**Table 2:** Simulation results for the population cause‐specific cumulative probabilities based on 500 simulated datasets with sample size of N={300,1000} for scenario 2. The performance measures are given for the nonparametric method (obtained via R‐package cmprsk) and for the flexible hazard‐based models (a), (b), and (c). Model (a) has a quadratic B‐spline baseline hazard function and knots at 1 and 5 years, whereas model (b) has a cubic B‐spline baseline hazard function with the same knots. The explanatory variables in all models were age and sex. Models (b) and (c) have the same baseline hazard function (cubic B‐spline with knots at 1 and 5 years). Models (a) and (b) have a fixed effect for sex, whereas model (c) has a TD effect for sex, which is modeled with a cubic B‐spline with two knots at 1 and 5 years

**Method**	**Cause**	**Time**	**True value**		**Relative**	**Bias(%)**	**empSE**		**RMSE**		**ModSE**		**Coverage** ^*†*^	
			*N* = 300	*N* = 1000	*N* = 300	*N* = 1000	*N* = 300	*N* = 1000	*N* = 300	*N* = 1000	*N* = 300	*N* = 1000	*N* = 300	*N* = 1000
		1	0.0967	0.0976	0.0450	0.7307	0.0172	0.0094	0.0172	0.0095	0.0173	0.0095	0.952	0.940
	1	5	0.3888	0.3937	−0.1937	−0.0651	0.0288	0.0157	0.0288	0.0157	0.0294	0.0161	0.956	0.958
Nonparametric		10	0.4762	0.4827	−0.1162	−0.2528	0.0310	0.0163	0.0310	0.0164	0.0309	0.0169	0.960	0.956
		1	0.0373	0.0363	1.5233	0.8476	0.0110	0.0060	0.0110	0.0060	0.0111	0.0060	0.950	0.966
	2	5	0.1366	0.1345	0.2634	0.6068	0.0198	0.0106	0.0198	0.0107	0.0207	0.0113	0.960	0.972
		10	0.2142	0.2135	0.1674	0.5309	0.0236	0.0135	0.0237	0.0135	0.0256	0.0140	0.968	0.962
		1	0.0967	0.0976	1.3942	1.7112	0.0149	0.0080	0.0150	0.0081	0.0147	0.0081	0.945	0.952
	1	5	0.3888	0.3937	0.0086	0.0678	0.0283	0.0155	0.0283	0.0155	0.0279	0.0154	0.943	0.962
Model (a)		10	0.4762	0.4827	−0.1155	−0.2633	0.0310	0.0164	0.0310	0.0164	0.0300	0.0166	0.952	0.952
		1	0.0373	0.0363	−0.1548	0.3552	0.0092	0.0050	0.0092	0.0050	0.0093	0.0050	0.956	0.937
	2	5	0.1366	0.1345	0.1740	0.6633	0.0186	0.0100	0.0186	0.0100	0.0185	0.0102	0.956	0.954
		10	0.2142	0.2135	−0.0256	0.3343	0.0237	0.0130	0.0237	0.0131	0.0238	0.0132	0.949	0.956
		1	0.0967	0.0976	1.4397	2.3070	0.0158	0.0087	0.0159	0.0090	0.0156	0.0086	0.950	0.937
	1	5	0.3888	0.3937	−0.0621	0.0660	0.0283	0.0154	0.0283	0.0154	0.0279	0.0154	0.944	0.958
Model (b)		10	0.4762	0.4827	−0.0970	−0.2620	0.0309	0.0163	0.0309	0.0163	0.0300	0.0166	0.954	0.952
		1	0.0373	0.0363	−0.1840	0.2910	0.0092	0.0050	0.0092	0.0050	0.0093	0.0050	0.954	0.937
	2	5	0.1366	0.1345	0.1730	0.6257	0.0187	0.0100	0.0187	0.0100	0.0185	0.0102	0.954	0.952
		10	0.2142	0.2135	0.0203	0.3489	0.0238	0.0131	0.0238	0.0132	0.0238	0.0132	0.948	0.954
		1	0.0967	0.0976	1.5913	2.3025	0.0159	0.0087	0.0160	0.0090	0.0156	0.0086	0.948	0.938
	1	5	0.3888	0.3937	−0.2547	0.0234	0.0284	0.0154	0.0284	0.0154	0.0279	0.0154	0.946	0.964
Model (c)		10	0.4762	0.4827	−0.1693	−0.2712	0.0308	0.0163	0.0308	0.0164	0.0301	0.0166	0.957	0.952
		1	0.0373	0.0363	−0.1159	0.3085	0.0093	0.0050	0.0093	0.0050	0.0093	0.0050	0.951	0.935
	2	5	0.1366	0.1345	0.1350	0.6176	0.0188	0.0100	0.0188	0.0101	0.0185	0.0102	0.948	0.952
		10	0.2142	0.2135	0.0687	0.4050	0.0239	0.0131	0.0239	0.0132	0.0238	0.0132	0.942	0.954

Abbreviations: empSE, empirical standard error; ModSE, model standard error; RMSE: root mean square error.

*†* Acceptable coverage range is [0.931,0.969] (calculated based on the work of Burton et al^30^)

We also tested whether a change in the knot location would affect the results. Therefore, we applied an additional model to each scenario, with the same specification as model (b) for scenario 1 and model (c) for scenario 2, but with the only difference being the knot location. The knots were corresponding to the 33rd and the 66th percentile of the time‐to‐cancer distribution, meaning that, for each dataset, there was a different set of two knots. The results for the new models (model (b^*′*^) and model (c^*′*^)) can be found in Table A1.3. Although differences compared to model (b) and model (c) were minor, we observed that the relative bias in the new models was in most cases less than 1% for each cause and at each time point, leading to more accurate results.

#### Performance on subgroups

**Figure 2:**
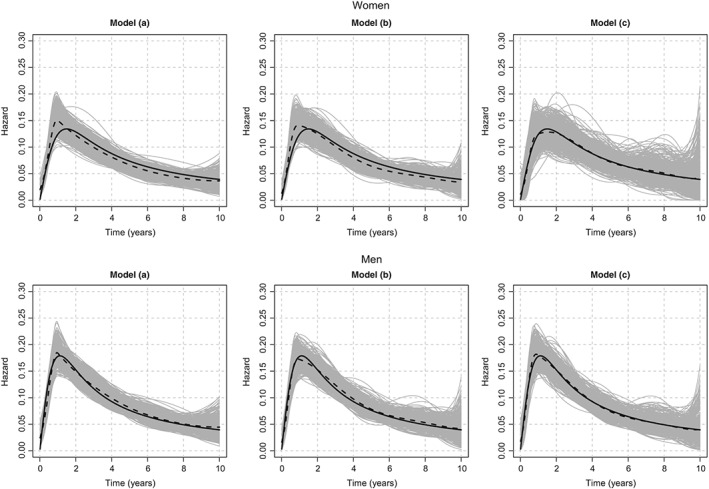
Simulated and estimated baseline hazard functions in scenario 2 with sample size of N=1000. In each panel, the bold solid curve represents the simulated baseline hazard function, the gray curves represent the 500 cause‐specific spline estimates, and the dashed curve represents the mean of these 500 estimates. Model (a) has a quadratic B‐spline baseline function with knots at 1 and 5 years, whereas model (b) has a cubic B‐spline baseline hazard function with the same knots. The explanatory variables in both models were age and sex. Models (b) and (c) have the same baseline function (cubic B‐spline with knots at 1 and 5 years). Models (a) and (b) have a fixed effect for sex, whereas model (c) has a TD effect for sex, which is modeled with a cubic B‐spline with two knots at 1 and 5 years

It is also interesting to look at the sex‐specific estimates and how these were affected by (i) model misspecification and (ii) the distribution of sex (men/women: 201/99 for *N*=300 and 703/297 for *N*=1000). Results found in Tables A1.1 and A1.2 show the performance of flexible parametric models separately in men and women. In the misspecified models (model (a) and model (b)), women were affected more than men especially in the earlier times regardless the sample size used. This was true for men only when the sample size was equal to *N*=1000. These results can be explained by the incapability of the models to estimate the baseline hazards adequately in the earlier times where a sudden peak occurred (see Figure A1.2 and Figure 2).

However, even with model (c), there was an occasion where the coverage probability at the 5th year for the 1st cause in case of women was slightly worse than expected when *N*=300 (Table A1.2). According to Figure A1.3, the estimates of the baseline hazard in the top right panel were rather unstable after the 4th year (although the median is in good agreement with the true), which can explain why we observed this poor coverage. With bigger sample sizes similar problems were not observed.

In summary, neglecting the TD effect for sex did not affect much the population estimates but it did affect the sex‐specific estimates. Also, the effect of sex estimated with model (b) was overestimated in the beginning and underestimated after 2 years, whereas with model (c), the estimated effect was approximating well the true one (see Figure A1.3).

## 4 ILLUSTRATIVE EXAMPLE

We used the mgus2 dataset from the R‐package survival to illustrate our approach. The dataset contains the time‐to‐occurrence of plasma cell malignancy (PCM) or death whichever comes first of individuals diagnosed with MGUS. By treating the progression to PCM as an absorbing state, we defined a competing risks setting that allowed subjects to make a single transition to one of two terminal states. Our goal was to estimate the cumulative probabilities of progressing to PCM and of death—while not having progressed to PCM—according to age at diagnosis (age), sex, and the size of the monoclonal serum splike (mspike). The R‐code to implement the following is explained and provided in Appendix 2.

From the 1384 people originally in the dataset, we removed 11 patients with missing values for the variable mspike. We observed 115 patients who progressed to PCM and 854 deaths among 627 females and 746 males. For each cause, we applied 8 models depending on whether time‐fixed or TD effects for each of the three variables were included, and the baseline hazard was modeled with a cubic spline with two knots located at the 33rd and the 66th percentile of the distribution of times to event (without distinguishing the type of event). We selected the best model using the Akaike Information Criterion. From the retained CSH regression models, we estimated the cumulative probability of progressing to PCM and the cumulative probability of death, and we compared those model‐based predicted probabilities to the nonparametric estimates, using the cuminc function of the R‐package cmprsk.

**Figure 3:**
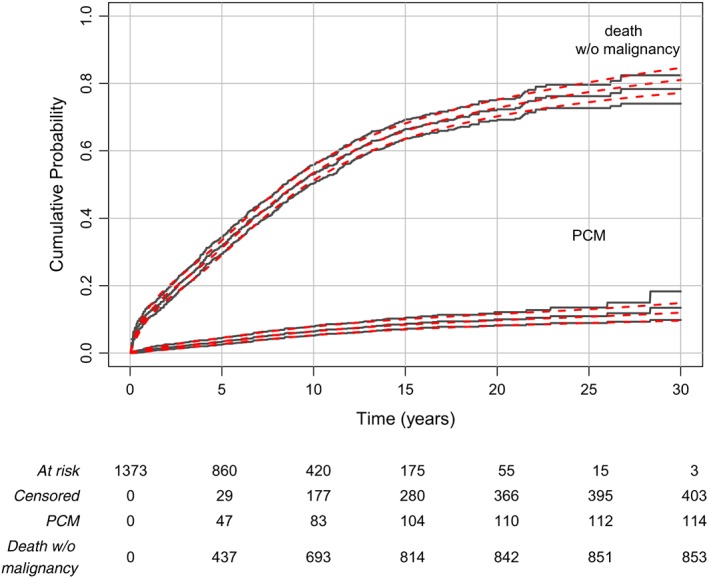
Cumulative probability of PCM and cumulative probability of death without malignancy over time (with the 95% confidence intervals), estimated using the nonparametric approach (solid lines) and our approach based on the flexible CSH models (dashed lines). The table below the graph indicated the number of subjects at risk as well as the cumulative number of each type of event [Colour figure can be viewed at wileyonlinelibrary.com]

**Figure 4:**
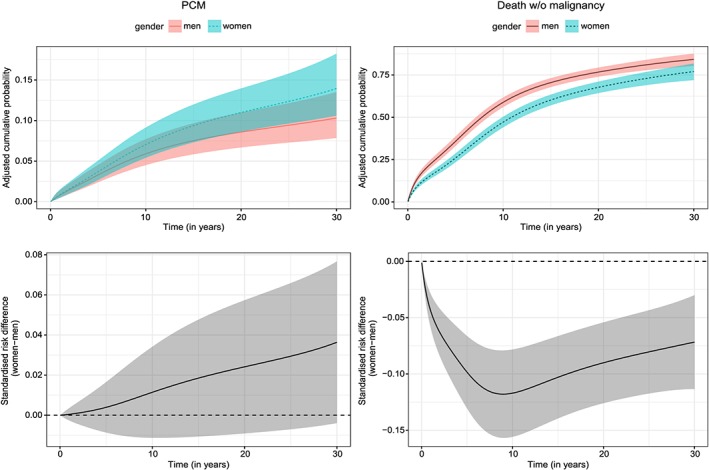
Adjusted cumulative probabilities of PCM (left top panel) and to death without malignancy (right top panel) for men and women, and standardized risk difference due to sex (women‐men) for PCM (left bottom panel) and death without malignancy (right bottom panel) [Colour figure can be viewed at wileyonlinelibrary.com]

For the event PCM, the selected model assumed a time‐fixed effect for the three variables, while for death a TD effect was retained only for age. The model‐based estimates of the cumulative probabilities of progressing to PCM and non‐PCM death are in very good agreement with the nonparametric estimates (Figure 3). We also quantified the effect of sex on the cumulative probability scale. To do so, two hypothetical populations were created, one where all patients were considered as women and another where all patients were considered as men, while keeping the other variables as observed. The new hypothetical populations had the same sample size with the initial dataset. We predicted the cumulative probabilities of progressing to PCM and non‐PCM death and plotted the probabilities for both populations along with the standardized risk difference due to sex for each cause (Figure 4). For progression to PCM, we observed a higher adjusted cumulative probability in women (7% (95% CI:[5;9]) vs 6% (95% CI:[5;8]) at 10 years and 14% (95% CI:[11;18]) vs 10% (95% CI:[8;14]) at 30 years), while for death, we observed that the adjusted cumulative probability was higher in men (59% (95% CI:[56;62]) vs 47% (95% CI:[44;50]) at 10 years and 84% (95% CI:[80;87]) vs 77% (95% CI:[71;82]) at 30 years). The corresponding time‐varying differences of adjusted cause‐specific probabilities between women and men are also displayed (Figure 4, bottom panels).

## 5 DISCUSSION

This work presented a way of estimating the cumulative incidence function through flexible regression models for the CSH. Once the regression coefficients of the CSH models have been estimated, one can derive individual or population estimates of the cause‐specific cumulative probabilities, with their corresponding variance accounting for the correlation between individuals predictions.^19^ We also showed how to derive directly adjusted cumulative probabilities for each competing event, in the same spirit of directly adjusted survival curves. Even though we exemplified our approach in the context of two CSHs, it may be easily extended to situation with more than two competing events.

In the competing risks setting, it is advised that we report both quantities due to the lack of one‐to‐one relationship between rates and risks.^31^ Indeed, a covariate may be strongly associated with one CSH while showing a fairly small effect on the cause‐specific cumulative probability (and vice versa). The cumulative probability for one cause is affected by the CSHs of all competing events and unlike subdistribution hazard models, we need to define the same number of models as the number of competing events observed in our data. On the other hand, the Fine and Gray approach based on subdistribution hazard modeling allows for a direct prediction of the cause‐specific cumulative probability although care is needed when interpreting the subdistribution hazard ratios.^10^ Using our approach and the directly adjusted cumulative probability, we can visualize and quantify (with the standardized risk difference) how the combination of the direct effect (on the CSH of interest) and the indirect effect (on the CSH of the competing event) of a given variable translates to the cumulative probability scale. It should be highlighted that CSH modeling in a competing risks analysis does not rely on the assumption that the competing risks are independent^3^; this assumption is not really needed for inference purposes.^1^

Flexible regression models for the CSH have been used to estimate the cumulative probabilities for different causes^12^ using models defined on the cumulative (cause‐specific) hazard scale that can accommodate both nonlinear (use of splines) and TD effects of covariates.^16^ We proposed in our approach to stick to the instantaneous hazard scale as we believe this scale is more familiar to researchers analyzing time‐to‐event data.^32^ Individual predictions of the cumulative probabilities can be derived using parameters estimated with regression models,^12^ and we extended here those predictions to population‐level estimates, while accounting for the correlation of individuals predictions obtained from the same set of regression parameters. Although nonparametric approaches are frequently used when population estimates are of interest, covariate effects on CSH and predictions for a given covariate pattern are additional advantages flexible regression models are bringing in.

We evaluated the ability of our approach in a simulation study and showed that our flexible model‐based approach performs well. We simulated two competing events using one specific distribution for cause 1 and demographic life tables for cause 2. Although we could have defined two parametric distributions (one for each cause), we decided to choose this specification in order to resemble a real data situation, ie, where *cancer* could be one of the causes following a known distribution and *other causes* is the competing event (coming from the general population mortality) to describe noncancer deaths. Our conclusions from the simulations are summarized as follows. Model estimates using cubic B‐splines outperformed the less flexible models (using quadratic B‐splines); they captured the shape of the cancer‐specific hazard better and gave more accurate estimates of the cumulative probabilities. Moreover, including a TD effect of sex gave better sex‐specific cumulative incidence estimates, while the omission of such TD effect was mainly impacting those shortly after the time of diagnosis (where the baseline hazard showed a rapid increase), but not considerably the overall population estimates. Comparing the time‐fixed and time‐varying cancer‐specific hazard ratios (Figure A1.3) confirmed this observation. Also, the multivariate delta method, used to derive the confidence intervals, provides a reliable method for in‐population estimation, with the requisite coverage probability properties. Regarding the sensitivity of the results to the number and position of the knots, previous work using empirical comparisons has shown that the cumulative probability is not affected by a sensible modification (eg, using the quantiles of the time‐to‐event distribution to define their location).^12^, ^33^ Indeed, we examined two additional models, which used the tertiles of the time‐to‐event distribution rather than fixed knots (at the 1st and 5th years). Using tertiles gave similar conclusions to the fixed knot model, with the only notable difference being in most cases a slight decrease in relative bias, when using tertiles.

When analyzing the simulated data, we adjusted for the covariates age at diagnosis and sex, which were both associated to the two CSHs used in the simulation. Thus, we did not use any model‐building strategy, and as far as we know, there is still no consensus regarding the “best” way to select the appropriate variable to adjust for, while accounting for complex nonlinear and TD effects.^34^^‐^ 36 Using a model‐building strategy for the CSH would call for refined methodology (such as bootstrap) when evaluating the variance of cumulative incidence functions.^37^^‐^ 39 Nevertheless, adjusting for confounders on the CSH of the competing event has been proved to be important even though the main interest is on the primary outcome.^26^ In the application, we relied on the Akaike Information Criteria to select the regression models for the CSHs, which provide the best fit to the data. We used cubic B‐splines for the baseline hazards with two internal knots corresponding to the 33rd and 66th percentiles. We obtained the smoothed cumulative incidence estimates that nicely matched the nonparametric ones. Using our approach, we also provided the adjusted cumulative probabilities of PCM and death in order to quantify the effect of sex in each cause, which would have been difficult to identify given the complicated nature of the cumulative probabilities.

In summary, our approach based on flexible CSH regression models demonstrated nice frequentist properties in estimating the cumulative probability for each cause. Estimation of the cumulative probabilities of death from each cause along with the CSH estimates provides a very useful insight of the underlying mechanisms of the competing events. We also presented a simple way of displaying and quantifying the overall (direct and indirect) impact of a variable on the cumulative probability of death from each cause through direct adjusted probability estimates. The R‐code associated with the R‐package mexhaz provides a tool in a free software, which could be useful to other researchers for computing these cumulative probabilities along with their confidence intervals (Appendix 2). It is worth noticing that the approach proposed here relies on the availability of the cause of death information. However, in many population‐based studies, the cause of death is either missing or unreliable. Thus, an adaptation of our approach to the relative survival setting^33^ (when reliable information on the cause of death is not available) would be an interesting extension.

## Supporting information

Figures A1.1‐A1.4 and Tables A1.1‐A1.3 can be found in Appendix 1. The R‐code is provided in Appendix 2.

SIM_8209‐Supp‐0001‐Appendix1.pdf

SIM_8209‐Supp‐0002‐Appendix2.pdf
