# Supplementary material for: Estimation of the adjusted cause‐specific cumulative probability using flexible regression models for the cause‐specific hazards
Source: Stat Med. 2019 Jun 18;38(20):3896–910. doi: 10.1002/sim.8209 (PMC6771712; doi:10.1002/sim.8209)
Supplement: Supplementary file 1 — SIM_8209‐Supp‐0001‐Appendix1.pdf [file SIM-38-3896-s001.pdf]

# Appendix 1

## Estimation of the adjusted cause-specific cumulative probability using flexible regression models for the cause-specific hazards

*DK Kipourou, H Charvat, B Rachet, A Belot*

## A Supplemetary results

Figure A1.1 : Simulated and estimated baseline hazard functions in *scenario 1* with sample size of $N = \{300, 1000\}$. In each panel, the bold solid curve represents the simulated baseline hazard function, the grey curves represent the 500 cause-specific spline estimates and the dashed curve represents the mean of the 500 estimates. Model (a) has a quadratic B-spline baseline hazard function and knots at 1 and 5 years. Models (b) has a cubic B-spline baseline function with the same knots. The explanatory variables in both models were age and sex.

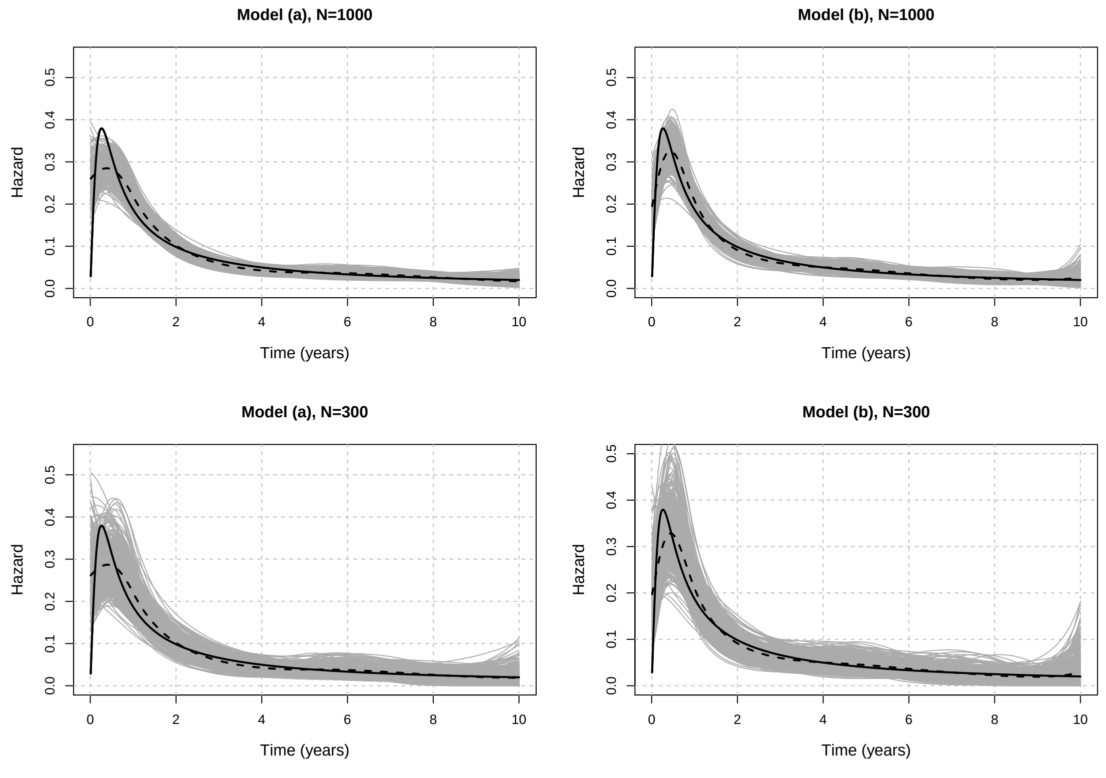

Figure A1.2 : Simulated and estimated baseline hazard functions in *scenario 2* with sample size of $N = 300$. In each panel, the bold solid curve represents the simulated baseline hazard function, the grey curves represent the 500 cause-specific spline estimates and the dashed curve represents the mean of these 500 estimates. Model (a) has a quadratic B-spline baseline hazard function with knots at 1 and 5 years, while model (b) has a cubic B-spline baseline hazard function with the same knots. The explanatory variables in both models were age and sex. Models (b) and (c) have the same baseline hazard function (cubic B-spline with knots at 1 and 5 years), but model (b) has a fixed effect for sex while model (c) has a time-dependent effect for sex, which is modelled with a cubic B-spline with two knots at 1 and 5 years.

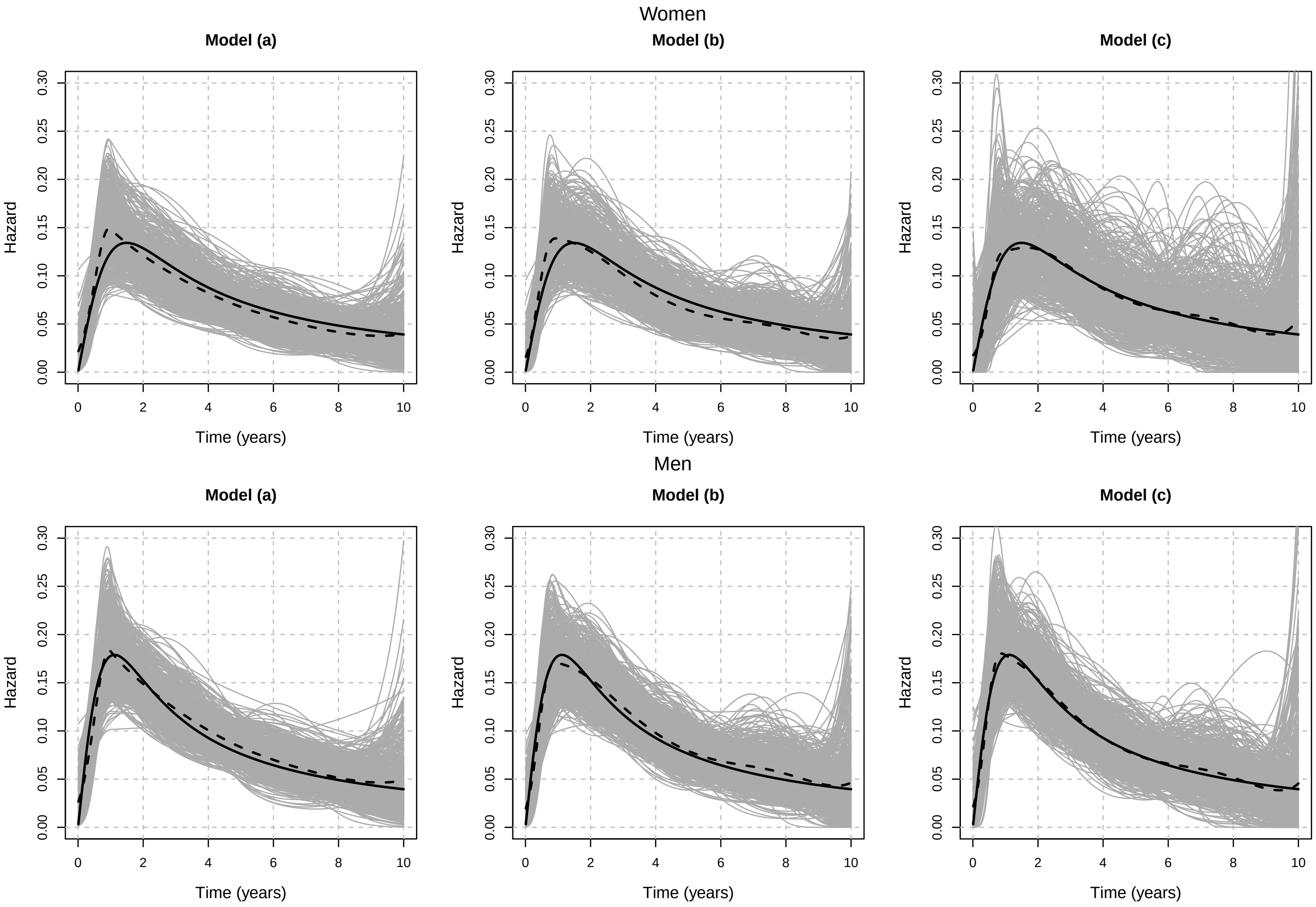

Figure A1.3 : Simulated and estimated time-dependent hazard ratio of sex provided for model (b) (PH) and model (c) (Non-PH) for *scenario 2*. Models (b) and (c) have the same baseline hazard function (cubic B-spline with knots at 1 and 5 years), but model (b) has a fixed effect for sex while model (c) has a time-dependent effect for sex, which is modelled with a cubic B-spline with two knots at 1 and 5 years. In each panel, the bold solid curve represents the simulated hazard ratio, the grey curves represent the 500 sample-specific cubic spline HR estimates, the dashed curve represents the median, and the dotted curve the mean of these 500 estimates.

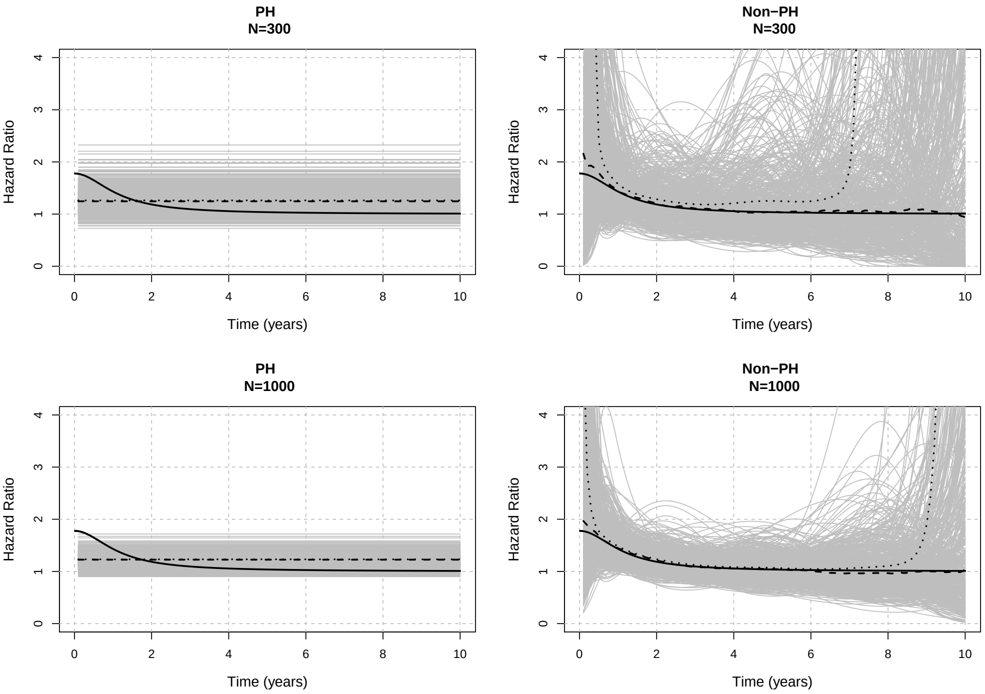

Figure A1.4 : Empirical distribution of the 500 parameter estimates of cumulative probabilities for each model and each cause at 3 timepoints: 1,5 and 10 years in *scenario 1*. Vertical lines denote the true values for each sample size setting $N = \{300, 1000\}$. Non-parametric estimates are provided using R-package `cmprsk` while model (a) and model (b) are flexible parametric models that are estimated using R-package `mexhaz`. Model (a) has a quadratic B-spline baseline hazard function and knots at 1 and 5 years, while model (b) has a cubic B-spline baseline function with the same knots. The explanatory variables in all models were age and sex.

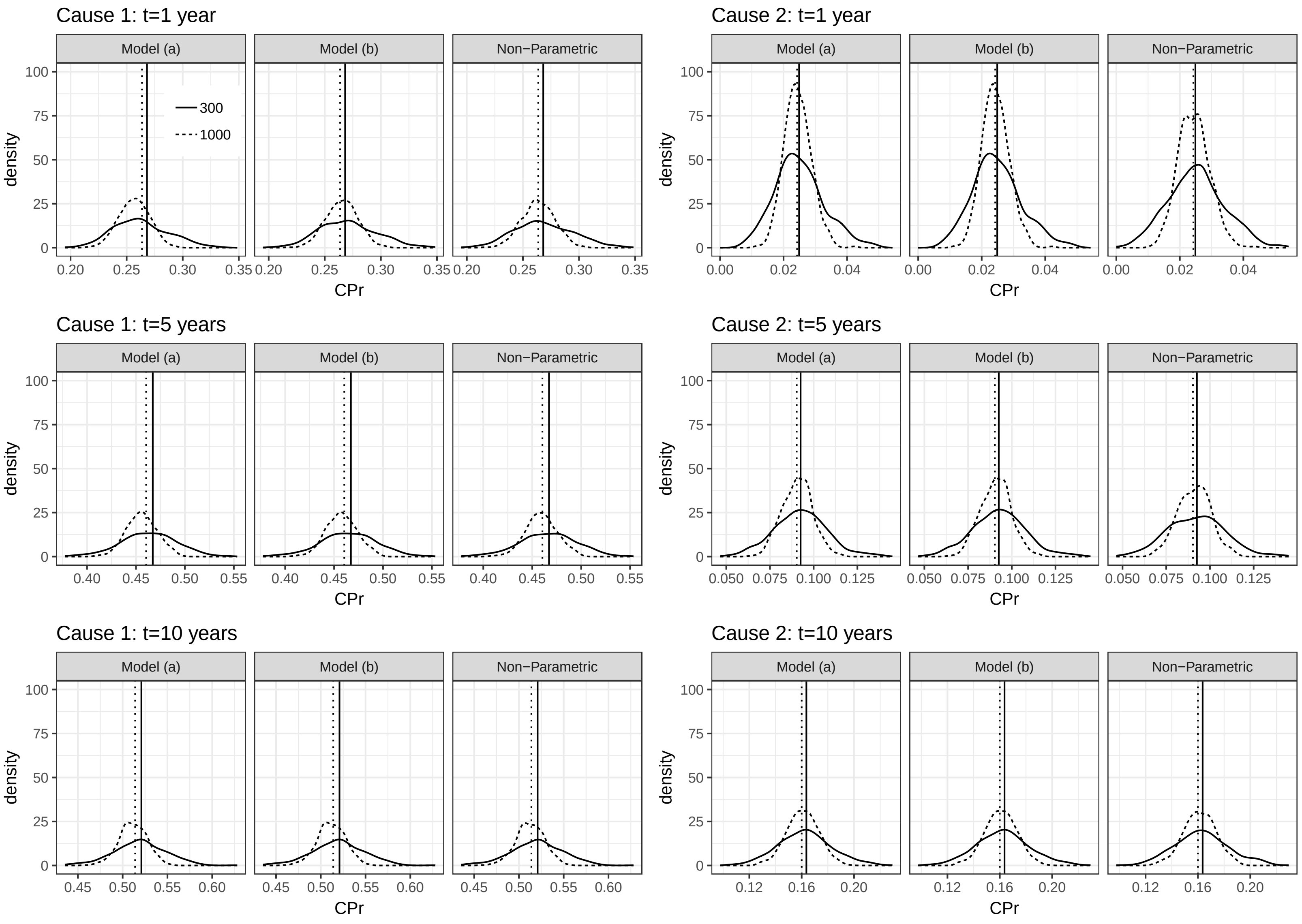

Table A1.1 : Performance results for the flexible parametric models regarding adjusted cumulative probabilities for *men* in *scenario 2*. The adjusted probabilities were obtained with the method described in Section 2.2.3. Model (a) has a quadratic B-spline baseline hazard function and knots at 1 and 5 years, while model (b) has a cubic B-spline baseline hazard function with the same knots. The explanatory variables in FPM models were age and sex. Models (b) and (c) have the same baseline hazard function (cubic B-spline with knots at 1 and 5 years), but model (b) has a fixed effect for sex while model (c) has a time-dependent effect for sex, which is modelled with a cubic B-spline with two knots at 1 and 5 years.

| **Method** | **Cause** | **Time** | **True value** | | **Mean value** | | **Bias***  | | **Relative Bias(%)** | | **empSE** | | **RMSE** | | **ModSE** | | **Coverage†** | |
|---|---|---|---|---|---|---|---|---|---|---|---|---|---|---|---|---|---|---|
| | | | N=300 | N=1000 | N=300 | N=1000 | N=300 | N=1000 | N=300 | N=1000 | N=300 | N=1000 | N=300 | N=1000 | N=300 | N=1000 | N=300 | N=1000 |
| Model (a) | 2 | 1 | 0.1102 | 0.1071 | 0.1050 | 0.1024 | -5.1802 | -4.6599 | -4.7025 | -4.3520 | 0.0170 | 0.0091 | 0.0178 | 0.0103 | 0.0166 | 0.0088 | 0.931 | 0.917 |
| | | 5 | 0.4072 | 0.4018 | 0.4076 | 0.4019 | 0.4697 | 0.0935 | 0.1154 | 0.0233 | 0.0337 | 0.0186 | 0.0338 | 0.0186 | 0.0338 | 0.0181 | 0.949 | 0.943 |
| | | 10 | 0.4886 | 0.4860 | 0.4933 | 0.4889 | 4.7230 | 2.8945 | 0.9666 | 0.5955 | 0.0369 | 0.0200 | 0.0372 | 0.0202 | 0.0364 | 0.0196 | 0.943 | 0.952 |
| | 2 | 1 | 0.0426 | 0.0385 | 0.0423 | 0.0382 | -0.2759 | -0.2337 | -0.6473 | -0.6075 | 0.0107 | 0.0054 | 0.0108 | 0.0054 | 0.0109 | 0.0055 | 0.960 | 0.949 |
| | | 5 | 0.1511 | 0.1387 | 0.1517 | 0.1394 | 0.5525 | 0.7239 | 0.3656 | 0.5220 | 0.0227 | 0.0116 | 0.0227 | 0.0117 | 0.0229 | 0.0118 | 0.954 | 0.945 |
| | | 10 | 0.2313 | 0.2161 | 0.2313 | 0.2166 | 0.0220 | 0.4720 | 0.0095 | 0.2184 | 0.0289 | 0.0154 | 0.0289 | 0.0155 | 0.0295 | 0.0156 | 0.943 | 0.947 |
| Model (b) | 1 | 1 | 0.1102 | 0.1071 | 0.1050 | 0.1030 | -5.1462 | -4.0601 | -4.6716 | -3.7919 | 0.0180 | 0.0099 | 0.0187 | 0.0107 | 0.0175 | 0.0093 | 0.935 | 0.911 |
| | | 5 | 0.4072 | 0.4018 | 0.4073 | 0.4019 | 0.1558 | 0.0877 | 0.0383 | 0.0218 | 0.0339 | 0.0186 | 0.0339 | 0.0186 | 0.0337 | 0.0181 | 0.950 | 0.947 |
| | | 10 | 0.4886 | 0.4860 | 0.4934 | 0.4889 | 4.7491 | 2.9025 | 0.9719 | 0.5972 | 0.0368 | 0.0200 | 0.0371 | 0.0202 | 0.0364 | 0.0196 | 0.944 | 0.952 |
| | 2 | 1 | 0.0426 | 0.0385 | 0.0423 | 0.0382 | -0.2911 | -0.2677 | -0.6830 | -0.6959 | 0.0107 | 0.0054 | 0.0107 | 0.0054 | 0.0109 | 0.0055 | 0.960 | 0.947 |
| | | 5 | 0.1511 | 0.1387 | 0.1517 | 0.1393 | 0.5464 | 0.6426 | 0.3615 | 0.4634 | 0.0227 | 0.0116 | 0.0227 | 0.0116 | 0.0229 | 0.0118 | 0.954 | 0.947 |
| | | 10 | 0.2313 | 0.2161 | 0.2314 | 0.2166 | 0.1438 | 0.4698 | 0.0622 | 0.2174 | 0.0289 | 0.0154 | 0.0289 | 0.0155 | 0.0295 | 0.0156 | 0.944 | 0.947 |
| Model (c) | 1 | 1 | 0.1102 | 0.1071 | 0.1121 | 0.1096 | 1.9203 | 2.4829 | 1.7432 | 2.3188 | 0.0206 | 0.0111 | 0.0207 | 0.0114 | 0.0203 | 0.0107 | 0.953 | 0.933 |
| | | 5 | 0.4072 | 0.4018 | 0.4073 | 0.4028 | 0.1565 | 0.9773 | 0.0384 | 0.2432 | 0.0345 | 0.0187 | 0.0345 | 0.0188 | 0.0344 | 0.0184 | 0.951 | 0.950 |
| | | 10 | 0.4886 | 0.4860 | 0.4888 | 0.4849 | 0.1556 | -1.1622 | 0.0319 | -0.2391 | 0.0364 | 0.0201 | 0.0364 | 0.0202 | 0.0367 | 0.0197 | 0.955 | 0.942 |
| | 2 | 1 | 0.0426 | 0.0385 | 0.0422 | 0.0381 | -0.4214 | -0.3834 | -0.9887 | -0.9967 | 0.0108 | 0.0054 | 0.0108 | 0.0054 | 0.0109 | 0.0055 | 0.959 | 0.948 |
| | | 5 | 0.1511 | 0.1387 | 0.1506 | 0.1383 | -0.5616 | -0.3262 | -0.3716 | -0.2352 | 0.0226 | 0.0115 | 0.0226 | 0.0115 | 0.0228 | 0.0118 | 0.957 | 0.946 |
| | | 10 | 0.2313 | 0.2161 | 0.2312 | 0.2165 | -0.1102 | 0.4075 | -0.0477 | 0.1886 | 0.0288 | 0.0154 | 0.0288 | 0.0154 | 0.0295 | 0.0156 | 0.944 | 0.950 |

empSE: empirical standard error; RMSE: root mean square error; ModSE: model standard error.

$*$ × 1000

† Acceptable coverage rate is $[0.931, 0.969]$ (calculated based on [30])

Table A1.2 : Performance results for the flexible parametric models regarding adjusted cumulative probabilities for *women* in *scenario 2*. The adjusted probabilities were obtained with the method described in Section 2.2.3. Model (a) has a quadratic B-spline baseline hazard function and knots at 1 and 5 years, while model (b) has a cubic B-spline baseline hazard function with the same knots. The explanatory variables in FPM models were age and sex. Models (b) and (c) have the same baseline hazard function (cubic B-spline with knots at 1 and 5 years). Models (a) and (b) have a fixed effect for sex while model (c) has a time-dependent effect for sex, which is modelled with a cubic B-spline with two knots at 1 and 5 years.

| **Method** | **Cause** | **Time** | **True value** | | **Mean value** | | **Bias***  | | **Relative Bias(%)** | | **empSE** | | **RMSE** | | **ModSE** | | **Coverage**† | |
|---|---|---|---|---|---|---|---|---|---|---|---|---|---|---|---|---|---|---|
| | | | N=300 | N=1000 | N=300 | N=1000 | N=300 | N=1000 | N=300 | N=1000 | N=300 | N=1000 | N=300 | N=1000 | N=300 | N=1000 | N=300 | N=1000 |
| Model (a) | 1 | 1 | 0.0693 | 0.0750 | 0.0839 | 0.0917 | 14.6018 | 16.6513 | 21.0717 | 22.1893 | 0.0169 | 0.0093 | 0.0223 | 0.0191 | 0.0167 | 0.0100 | 0.840 | 0.574 |
| | | 5 | 0.3515 | 0.3744 | 0.3506 | 0.3750 | -0.8522 | 0.6770 | -0.2425 | 0.1809 | 0.0462 | 0.0260 | 0.0462 | 0.0261 | 0.0441 | 0.0261 | 0.935 | 0.949 |
| | | 10 | 0.4510 | 0.4748 | 0.4398 | 0.4637 | -11.2564 | -11.1302 | -2.4957 | -2.3442 | 0.0525 | 0.0286 | 0.0537 | 0.0307 | 0.0499 | 0.0292 | 0.945 | 0.935 |
| | 2 | 1 | 0.0266 | 0.0311 | 0.0270 | 0.0320 | 0.3850 | 0.9868 | 1.4464 | 3.1777 | 0.0090 | 0.0058 | 0.0090 | 0.0059 | 0.0092 | 0.0058 | 0.945 | 0.933 |
| | | 5 | 0.1072 | 0.1247 | 0.1068 | 0.1260 | -0.4015 | 1.2912 | -0.3745 | 1.0352 | 0.0251 | 0.0156 | 0.0251 | 0.0156 | 0.0258 | 0.0163 | 0.952 | 0.943 |
| | | 10 | 0.1793 | 0.2075 | 0.1791 | 0.2088 | -0.2105 | 1.2862 | -0.1174 | 0.6200 | 0.0370 | 0.0228 | 0.0370 | 0.0228 | 0.0377 | 0.0236 | 0.954 | 0.956 |
| Model (b) | 1 | 1 | 0.0693 | 0.0750 | 0.0840 | 0.0922 | 14.6660 | 17.1886 | 21.1643 | 22.9054 | 0.0174 | 0.0098 | 0.0228 | 0.0198 | 0.0174 | 0.0104 | 0.839 | 0.590 |
| | | 5 | 0.3515 | 0.3744 | 0.3504 | 0.3750 | -1.0478 | 0.6675 | -0.2981 | 0.1783 | 0.0458 | 0.0259 | 0.0459 | 0.0260 | 0.0440 | 0.0261 | 0.935 | 0.949 |
| | | 10 | 0.4510 | 0.4748 | 0.4400 | 0.4637 | -11.0414 | -11.1286 | -2.4480 | -2.3439 | 0.0521 | 0.0286 | 0.0533 | 0.0307 | 0.0499 | 0.0293 | 0.948 | 0.935 |
| | 2 | 1 | 0.0266 | 0.0311 | 0.0270 | 0.0320 | 0.3829 | 0.9889 | 1.4382 | 3.1843 | 0.0090 | 0.0059 | 0.0090 | 0.0059 | 0.0092 | 0.0058 | 0.948 | 0.933 |
| | | 5 | 0.1072 | 0.1247 | 0.1068 | 0.1260 | -0.3931 | 1.3134 | -0.3666 | 1.0529 | 0.0253 | 0.0158 | 0.0254 | 0.0159 | 0.0258 | 0.0163 | 0.948 | 0.941 |
| | | 10 | 0.1793 | 0.2075 | 0.1792 | 0.2089 | -0.1601 | 1.3966 | -0.0893 | 0.6732 | 0.0372 | 0.0231 | 0.0372 | 0.0231 | 0.0377 | 0.0236 | 0.952 | 0.954 |
| Model (c) | 1 | 1 | 0.0693 | 0.0750 | 0.0701 | 0.0767 | 0.7628 | 1.6864 | 1.1008 | 2.2472 | 0.0221 | 0.0141 | 0.0221 | 0.0142 | 0.0229 | 0.0139 | 0.957 | 0.933 |
| | | 5 | 0.3515 | 0.3744 | 0.3481 | 0.3724 | -3.3183 | -2.0029 | -0.9441 | -0.5350 | 0.0513 | 0.0276 | 0.0514 | 0.0277 | 0.0468 | 0.0277 | 0.927 | 0.956 |
| | | 10 | 0.4510 | 0.4748 | 0.4483 | 0.4731 | -2.7595 | -1.6572 | -0.6118 | -0.3490 | 0.0557 | 0.0307 | 0.0557 | 0.0307 | 0.0516 | 0.0303 | 0.944 | 0.942 |
| | 2 | 1 | 0.0266 | 0.0311 | 0.0273 | 0.0323 | 0.7245 | 1.2841 | 2.7216 | 4.1351 | 0.0092 | 0.0059 | 0.0092 | 0.0060 | 0.0093 | 0.0059 | 0.944 | 0.931 |
| | | 5 | 0.1072 | 0.1247 | 0.1089 | 0.1283 | 1.6992 | 3.5697 | 1.5850 | 2.8618 | 0.0260 | 0.0160 | 0.0261 | 0.0164 | 0.0263 | 0.0166 | 0.948 | 0.931 |
| | | 10 | 0.1793 | 0.2075 | 0.1800 | 0.2094 | 0.6694 | 1.9476 | 0.3733 | 0.9387 | 0.0376 | 0.0231 | 0.0376 | 0.0232 | 0.0378 | 0.0237 | 0.948 | 0.952 |

empSE: empirical standard error; RMSE: root mean square error; ModSE: model standard error.

$* \times 1000$

† Acceptable coverage rate is $[0.931, 0.969]$ (calculated based on [30])

Table A1.3 : Simulation results for the population cause-specific cumulative probabilities based on 500 simulated datasets with sample size of $N = \{300, 1000\}$ for *scenario 1* and *scenario 2*. The performance measures are given for the flexible parametric models (model (b′) and model (c′)). Model (b′) has a quadratic B-spline baseline hazard function, while model (c′) has a cubic B-spline baseline hazard function including a time-dependent effect for sex. In both models, we used two knots corresponding to the 33rd and the 66th percentile of the time-to-cancer distribution of each dataset. The explanatory variables in both models were age and sex.

| **Scenario/ Model** | **Cause** | **Time** | **True value** | | **Mean value** | | **Bias*** | | **Relative Bias(%)** | | **empSE** | | **RMSE** | | **ModSE** | | **Coverage†** | |
|---|---|---|---|---|---|---|---|---|---|---|---|---|---|---|---|---|---|---|
| | | | N=300 | N=1000 | N=300 | N=1000 | N=300 | N=1000 | N=300 | N=1000 | N=300 | N=1000 | N=300 | N=1000 | N=300 | N=1000 | N=300 | N=1000 |
| Scenario 1/ Model (b′) | 1 | 1 | 0.2681 | 0.2637 | 0.2693 | 0.2635 | 1.1511 | -0.2426 | 0.4293 | -0.0920 | 0.0239 | 0.0131 | 0.0239 | 0.0131 | 0.0239 | 0.0130 | 0.9516 | 0.9577 |
| | | 5 | 0.4672 | 0.4604 | 0.4662 | 0.4588 | -1.0151 | -1.5622 | -0.2173 | -0.3393 | 0.0288 | 0.0155 | 0.0288 | 0.0156 | 0.0284 | 0.0156 | 0.9456 | 0.9598 |
| | | 10 | 0.5209 | 0.5139 | 0.5195 | 0.5121 | -1.3477 | -1.8817 | -0.2587 | -0.3661 | 0.0293 | 0.0156 | 0.0294 | 0.0157 | 0.0295 | 0.0162 | 0.9476 | 0.9598 |
| | 2 | 1 | 0.0249 | 0.0243 | 0.0248 | 0.0243 | -0.0737 | 0.0054 | -0.2961 | 0.0221 | 0.0078 | 0.0042 | 0.0078 | 0.0042 | 0.0077 | 0.0042 | 0.9456 | 0.9497 |
| | | 5 | 0.0925 | 0.0903 | 0.0926 | 0.0908 | 0.0778 | 0.4860 | 0.0841 | 0.5381 | 0.0153 | 0.0087 | 0.0153 | 0.0087 | 0.0160 | 0.0087 | 0.9556 | 0.9557 |
| | | 10 | 0.1636 | 0.1600 | 0.1627 | 0.1610 | -0.8965 | 0.9696 | -0.5480 | 0.6060 | 0.0206 | 0.0121 | 0.0206 | 0.0122 | 0.0222 | 0.0122 | 0.9597 | 0.9396 |
| Scenario 2/ Model (c′) | 1 | 1 | 0.0967 | 0.0976 | 0.0958 | 0.0979 | -0.8300 | 0.3191 | -0.8585 | 0.3271 | 0.0166 | 0.0093 | 0.0156 | 0.0093 | 0.0167 | 0.0087 | 0.9437 | 0.9276 |
| | | 5 | 0.3888 | 0.3937 | 0.3877 | 0.3934 | -1.0513 | -0.2342 | -0.2704 | -0.0595 | 0.0284 | 0.0155 | 0.0280 | 0.0155 | 0.0284 | 0.0155 | 0.9457 | 0.9618 |
| | | 10 | 0.4762 | 0.4827 | 0.4755 | 0.4813 | -0.6653 | -1.3560 | -0.1397 | -0.2809 | 0.0309 | 0.0163 | 0.0301 | 0.0164 | 0.0309 | 0.0166 | 0.9557 | 0.9517 |
| | 2 | 1 | 0.0373 | 0.0363 | 0.0373 | 0.0364 | -0.0809 | 0.1444 | -0.2167 | 0.3981 | 0.0092 | 0.0050 | 0.0092 | 0.0050 | 0.0092 | 0.0050 | 0.9557 | 0.9376 |
| | | 5 | 0.1366 | 0.1345 | 0.1367 | 0.1354 | 0.1142 | 0.8733 | 0.0836 | 0.6491 | 0.0186 | 0.0100 | 0.0185 | 0.0101 | 0.0186 | 0.0102 | 0.9517 | 0.9517 |
| | | 10 | 0.2142 | 0.2135 | 0.2143 | 0.2144 | 0.1381 | 0.8514 | 0.0645 | 0.3987 | 0.0237 | 0.0131 | 0.0238 | 0.0132 | 0.0237 | 0.0132 | 0.9457 | 0.9537 |

empSE: empirical standard error; RMSE: root mean square error; ModSE: model standard error.

$*$ $\times$ 1000

† Acceptable coverage rate is $[0.931, 0.969]$ (calculated based on [30])

# B Details for variance estimation of the crude probabilities

The partial derivative of $F_j$ with respect to the parameter $\beta_i$ is given by

$$\begin{aligned}\frac{\partial F_j(t,\mathbf{x};\boldsymbol{\beta})}{\partial \beta_i} =& \frac{\partial}{\partial \beta_i}\Big[\int_0^t S_j(u,\mathbf{x};\boldsymbol{\beta})S_{\bar{j}}(u,\mathbf{x};\boldsymbol{\beta})\lambda_j(u,\mathbf{x};\boldsymbol{\beta})\mathrm{d}u\Big] \\ =& \int_0^t \frac{\partial S_j(u,\mathbf{x};\boldsymbol{\beta})}{\partial \beta_i} S_{\bar{j}}(u,\mathbf{x};\boldsymbol{\beta})\lambda_j(u,\mathbf{x};\boldsymbol{\beta})\mathrm{d}u \\ &+ \int_0^t S_j(u,\mathbf{x};\boldsymbol{\beta})\frac{\partial S_{\bar{j}}(u,\mathbf{x};\boldsymbol{\beta})}{\partial \beta_i}\lambda_j(u,\mathbf{x};\boldsymbol{\beta})\mathrm{d}u \\ &+ \int_0^t S_j(u,\mathbf{x};\boldsymbol{\beta})S_{\bar{j}}(u,\mathbf{x};\boldsymbol{\beta})\frac{\partial \lambda_j(u,\mathbf{x};\boldsymbol{\beta})}{\partial \beta_i}\mathrm{d}u\end{aligned} \tag{1}$$

Note that the corresponding formula for $F_{\bar{j}}$ is obtained by exchanging the roles of $j$ and $\bar{j}$ in the above expression.

The partial derivative of $S_j$ with respect to $\beta_i$ is

$$\begin{aligned}\frac{\partial S_j(t,\mathbf{x};\boldsymbol{\beta})}{\partial \beta_i} &= \frac{\partial}{\partial \beta_i}\Big[\exp(-\int_0^t \lambda_j(u,\mathbf{x};\boldsymbol{\beta})\mathrm{d}u)\Big] \\ &= \frac{\partial}{\partial \beta_i}\Big[-\int_0^t \lambda_j(u,\mathbf{x};\boldsymbol{\beta})\mathrm{d}u\Big]S_j(t,\mathbf{x};\boldsymbol{\beta}) \\ &= -S_j(t,\mathbf{x};\boldsymbol{\beta})\int_0^t \frac{\partial \lambda_j(u,\mathbf{x};\boldsymbol{\beta})}{\partial \beta_i}\mathrm{d}u\end{aligned} \tag{2}$$

if $\beta_i \in \boldsymbol{\beta}_j$ and 0 otherwise.

From formulae 1 and 2, it follows that

$$\begin{aligned}\frac{\partial F_j(t,\mathbf{x};\boldsymbol{\beta})}{\partial \beta_i} &= \int_0^t S(u,\mathbf{x};\boldsymbol{\beta})\left(-\int_0^u \left(\frac{\partial \lambda_j(v,\mathbf{x};\boldsymbol{\beta})}{\partial \beta_i} + \frac{\partial \lambda_{\bar{j}}(v,\mathbf{x};\boldsymbol{\beta})}{\partial \beta_i}\right)\mathrm{d}v\right)\lambda_j(u,\mathbf{x};\boldsymbol{\beta})\mathrm{d}u + \int_0^t S(u,\mathbf{x};\boldsymbol{\beta})\frac{\partial \lambda_j(u,\mathbf{x};\boldsymbol{\beta})}{\partial \beta_i}\mathrm{d}u \\ &= \int_0^t S(u,\mathbf{x};\boldsymbol{\beta})\left(\frac{\partial \lambda_j(u,\mathbf{x};\boldsymbol{\beta})}{\partial \beta_i} - \lambda_j(u,\mathbf{x};\boldsymbol{\beta})\int_0^u \left(\frac{\partial \lambda_j(v,\mathbf{x};\boldsymbol{\beta})}{\partial \beta_i} + \frac{\partial \lambda_{\bar{j}}(v,\mathbf{x};\boldsymbol{\beta})}{\partial \beta_i}\right)\mathrm{d}v\right)\mathrm{d}u\end{aligned} \tag{3}$$

In our work, we used flexible regression models defined on the log-hazard scale. Thus, the $\lambda_j$s may be written as exponentials of differentiable functions $P_j$, *i.e.*, $\lambda_j(t,\mathbf{x};\boldsymbol{\beta}) = \exp\big(P_j(t,\mathbf{x};\boldsymbol{\beta})\big)$, which leads to

$$\frac{\partial \lambda_j(t,\mathbf{x};\boldsymbol{\beta})}{\partial \beta_i} = \frac{\partial}{\partial \beta_i}\big[\exp\left(P_j(t,\mathbf{x};\boldsymbol{\beta})\right)\big] = \lambda_j(t,\mathbf{x};\boldsymbol{\beta})\frac{\partial P_j(t,\mathbf{x};\boldsymbol{\beta})}{\partial \beta_i}$$

Formula 3 can now be written

$$\begin{aligned}\frac{\partial F_j(t,\mathbf{x};\boldsymbol{\beta})}{\partial \beta_i} &= \int_0^t S(u,\mathbf{x};\boldsymbol{\beta})\left(\lambda_j(u,\mathbf{x};\boldsymbol{\beta})\frac{\partial P_j(u,\mathbf{x};\boldsymbol{\beta})}{\partial \beta_i} - \lambda_j(u,\mathbf{x};\boldsymbol{\beta})\int_0^u \left(\lambda_j(v,\mathbf{x};\boldsymbol{\beta})\frac{\partial P_j(v,\mathbf{x};\boldsymbol{\beta})}{\partial \beta_i} + \lambda_{\bar{j}}(v,\mathbf{x};\boldsymbol{\beta})\frac{\partial P_{\bar{j}}(v,\mathbf{x};\boldsymbol{\beta})}{\partial \beta_i}\right)\mathrm{d}v\right)\mathrm{d}u \\ &= \int_0^t S(u,\mathbf{x};\boldsymbol{\beta})\lambda_j(u,\mathbf{x};\boldsymbol{\beta})\left(\frac{\partial P_j(u,\mathbf{x};\boldsymbol{\beta})}{\partial \beta_i} - \int_0^u \left(\lambda_j(v,\mathbf{x};\boldsymbol{\beta})\frac{\partial P_j(v,\mathbf{x};\boldsymbol{\beta})}{\partial \beta_i} + \lambda_{\bar{j}}(v,\mathbf{x};\boldsymbol{\beta})\frac{\partial P_{\bar{j}}(v,\mathbf{x};\boldsymbol{\beta})}{\partial \beta_i}\right)\mathrm{d}v\right)\mathrm{d}u \\ &= \begin{cases}\displaystyle\int_0^t S(u,\mathbf{x};\boldsymbol{\beta})\lambda_j(u,\mathbf{x};\boldsymbol{\beta})\left(\frac{\partial P_j(u,\mathbf{x};\boldsymbol{\beta})}{\partial \beta_i} - \int_0^u \lambda_j(v,\mathbf{x};\boldsymbol{\beta})\frac{\partial P_j(v,\mathbf{x};\boldsymbol{\beta})}{\partial \beta_i}\mathrm{d}v\right)\mathrm{d}u & ,\beta_i \in \boldsymbol{\beta}_j \\ \displaystyle\int_0^t S(u,\mathbf{x};\boldsymbol{\beta})\lambda_j(u,\mathbf{x};\boldsymbol{\beta})\left(-\int_0^u \lambda_{\bar{j}}(v,\mathbf{x};\boldsymbol{\beta})\frac{\partial P_{\bar{j}}(v,\mathbf{x};\boldsymbol{\beta})}{\partial \beta_i}\mathrm{d}v\right)\mathrm{d}u & ,\text{otherwise}\end{cases}\end{aligned} \tag{4}$$

# C Estimation of the variance of the difference between adjusted probabilities

Following the reasoning in section 2.2.2, the variance of the difference between the adjusted probabilities of death with different treatments, say A and B, is given by

$$Var[D(t;\boldsymbol{\beta})] = \mathbf{w}^\top [\nabla D(t;\boldsymbol{\beta})^{\mathrm{Mat}}]^\top_{|\boldsymbol{\beta}=\widehat{\boldsymbol{\beta}}} \widehat{\boldsymbol{\Sigma}}_{\boldsymbol{\beta}} [\nabla D(t;\boldsymbol{\beta})^{\mathrm{Mat}}]_{|\boldsymbol{\beta}=\widehat{\boldsymbol{\beta}}} \mathbf{w}$$

where $\nabla D(t;\boldsymbol{\beta})^{\mathrm{Mat}}$ is a $(m \times N)$ matrix:

$$\begin{aligned}
\nabla D(t;\boldsymbol{\beta})^{\mathrm{Mat}} &= \left(\nabla F_j^{\mathrm{Mat}}(t,A;\boldsymbol{\beta}) - \nabla F_j^{\mathrm{Mat}}(t,B;\boldsymbol{\beta})\right) \\
&= \left(\left(\nabla F_j(t,A,\mathbf{z}_1;\boldsymbol{\beta})_{|\boldsymbol{\beta}=\widehat{\boldsymbol{\beta}}}, \cdots, \nabla F_j(t,A,\mathbf{z}_N;\boldsymbol{\beta})_{|\boldsymbol{\beta}=\widehat{\boldsymbol{\beta}}}\right) - \left(\nabla F_j(t,B,\mathbf{z}_1;\boldsymbol{\beta})_{|\boldsymbol{\beta}=\widehat{\boldsymbol{\beta}}}, \cdots, \nabla F_j(t,B,\mathbf{z}_N;\boldsymbol{\beta})_{|\boldsymbol{\beta}=\widehat{\boldsymbol{\beta}}}\right)\right) \\
&= \left(\nabla F_j\left(t,A,\mathbf{z}_1;\boldsymbol{\beta}\right)_{|\boldsymbol{\beta}=\widehat{\boldsymbol{\beta}}} - \nabla F_j\left(t,B,\mathbf{z}_1;\boldsymbol{\beta}\right)_{|\boldsymbol{\beta}=\widehat{\boldsymbol{\beta}}}, \cdots, \nabla F_j(t,A,\mathbf{z}_N;\boldsymbol{\beta})_{|\boldsymbol{\beta}=\widehat{\boldsymbol{\beta}}} - \nabla F_j(t,B,\mathbf{z}_N;\boldsymbol{\beta})_{|\boldsymbol{\beta}=\widehat{\boldsymbol{\beta}}}\right)
\end{aligned}$$

and $w = \frac{1}{N}$.
