# Supplementary material for: Estimation of the adjusted cause‐specific cumulative probability using flexible regression models for the cause‐specific hazards
Source: Stat Med. 2019 Jun 18;38(20):3896–910. doi: 10.1002/sim.8209 (PMC6771712; doi:10.1002/sim.8209)
Supplement: Supplementary file 2 — SIM_8209‐Supp‐0002‐Appendix2.pdf [file SIM-38-3896-s002.pdf]

Appendix 2

Estimation of cause -specific cumulative probabilities in competing risks setting using flexible hazard-based regression models fitted with the R-package `mexhaz`

DK Kipourou, H Charvat, B Rachet, A Belot

This tutorial describes the necessary steps to estimate the event-specific cumulative probabilities after fitting a cause-specific (or more generally, event-specific) flexible hazard-based regression models, using the R-package `mexhaz`. The estimation of the (adjusted) event-specific cumulative probabilities and at the population level is a 3-step procedure:

1. Manage the data
2. Fit the flexible hazard-based regression models for each event
3. Estimate the (adjusted) event-specific cumulative probabilities and the standardised risk differences

Before detailing the steps, we start by describing how to set the R-environment for our objective. We need the following R-packages: `Matrix`, `mexhaz`, `plyr`, and `doSNOW`; all but the last, are used for the estimations, while `doSNOW` is used for parallel computing. We will also use the packages `ggplot2` and `gridExtra` to plot some results.

Additionally, before going into any calculations we need to run the `CumIncid.R` which contains all the essential functions for this tutorial. A copy of the script can be found at the end of the tutorial.

One important thing for being able to follow this tutorial is to check the version of the R-package `mexhaz`, as it works currently with **version "1.5"**. Moreover, you need to set the working directory to the folder where the file `CumIncid.R` has been saved ("myWorkingfolder").

```r
# Change this path appropriately
#setwd("your path")

# Install the needed packages
reqPcks <- c("doSNOW", "mexhaz","Matrix","ggplot2", "plyr", "gridExtra")

for(p in reqPcks){
  if(!require(p, character.only=TRUE)) {
    install.packages(p)
    library(p, character.only = TRUE)}
}
```

1 Data management

We use the `mgus2` dataset from the R-package `survival`. The dataset contains the time to the occurrence of plasma cell malignancy (PCM) or death (whichever comes first), of people

diagnosed with monoclonal gammopathy of undetermined significance (MGUS). By treating progression to PCM as an absorbing state we defined a competing risks setting that allowed subjects to make a single transition to one of two terminal states. Our goal was to estimate the cumulative probabilities of progressing to PCM and of death -while not having progressed to PCM-, according to age at diagnosis (age), sex, and the size of the monoclonal serum spike (mspike).

```
#Data
#-----

# Load the data
data("mgus2",package="survival")
mydata <- mgus2

# Create variable <timesurv> with the time to the first event (years)
mydata$timesurv <- with(mydata, ifelse(pstat==0, futime, ptime))
mydata$timesurv <- mydata$timesurv/12

# Create variable <event> defining the type of the first event
mydata$event <- with(mydata, ifelse(pstat==0, 2*death, 1))
mydata$event <- factor(mydata$event, levels=0:2,
                       labels=c("censor","pcm","death"))

# Create a variable <agec>, centered at 70 years old
mydata$agec <- mydata$age-70

# Discard missing values from <mspike>
mydata <- mydata[is.na(mydata$mspike)==F,]


dim(mydata)

## [1] 1373   13

table(mydata$event)

##
## censor    pcm  death
##    404    115    854
```

We create a new variable defining the time to the first event measured in years (`timesurv`), and a variable (`event`) defining the type of the first observed event, including censoring as a "type" of event (censor, PCM, and death). We exclude observations with missing values for `mspike` (11 observations). Among the 1373 individuals, we observed 115 occurrences of PCM events and 854 deaths (as the first event, *i.e.* without occurrence of PCM), while 404 patients were censored alive without PCM.

2 Fit of the flexible hazard-based regression models for each event

We fitted 2 flexible regression models, one for each type of event. To select the regression model, we fitted 8 models depending on whether time-fixed or time-dependent effects for each

of the 3 variables were included. The baseline hazard was modelled with a cubic spline with 2 knots located at the 33rd and the 66th percentile of the times to event distribution (without distinguishing the type of event). We selected the best model using the Akaike Loormation Criterion. For the event PCM, the selected model assumed a time-fixed effect for the 3 variables, while for death a time-dependent effect was retained for age. We report here only the fit of the finally selected model for each event.

```
# myk defines the location of the knots
(myk <- quantile(mydata$timesurv, probs=c(1/3,2/3)))

## 33.33333% 66.66667%
##  4.416667  9.388889

# Run flexible parametric models for each event (Mod1 for pcm, Mod2 for death)
Mod1 <- mexhaz(Surv(timesurv,event=="pcm") ~ agec + mspike + sex,
               data = mydata, base = "exp.bs", degree = 3, knots = myk,
               verbose = 0, print.level = 0)

## Computation of the Hessian
##
## Data
##    Name N.Obs.Tot N.Obs N.Events N.Clust
##  mydata      1373  1373      115       1
##
## Details
##  Iter Eval   Base Nb.Leg Nb.Aghq Optim Method Code
##   124 1153 exp.bs     20      10   nlm    ---    1
##     LogLik Total.Time
##  -617.2745       2.86

Mod2 <- mexhaz(Surv(timesurv, event=="death") ~ agec + nph(agec) +
               mspike + sex, data = mydata, base = "exp.bs",
               degree = 3, knots = myk, verbose = 0, print.level = 0)

## Computation of the Hessian
##
## Data
##    Name N.Obs.Tot N.Obs N.Events N.Clust
##  mydata      1373  1373      854       1
##
## Details
##  Iter Eval   Base Nb.Leg Nb.Aghq Optim Method Code
##   151 1558 exp.bs     20      10   nlm    ---    1
##     LogLik Total.Time
##  -2767.594       9.36
```

3 Estimation of the event-specific cumulative probabilities

3.1 Main functions to be used

In this section, we will list the main functions needed in order to estimate the event-specific cumulative probabilities (point estimates and 95% confidence intervals) along with other useful

quantities. Below follows a brief explanation of the basic functions while a full specification of them can be found in the `CumIncid.R`, in the end of this document.

1. `csprob(data_df, modA, modB, time.max, frag)`

   Function that estimates the event-specific cumulative probabilities for all the individuals included in the `data_df`. The results are based on the event-specific regression models that should be defined in the function as `modA` and `modB` (event-specific models corresponding to 2 events). The `time.max` is the maximum time for which we want to estimate the event-specific cumulative probabilities, `subdiv` is the number of subintervals that the interval [0, time.max] should be split into. The output of this function is a list each element of which contains the individual-specific results.

2. `predict.prob(csprobObj, pop)`

   Function that uses the quantities that are calculated with the `csprob` function (`csprobObj`) and predicts the event-specific probabilities for a fine grid of time-points, for either specific covariates or for the whole population. For the population estimates and confidence intervals, `pop` should be set to `TRUE`. If `pop=FALSE`, then in the results we will have all the covariate-specific point estimates and 95% confidence intervals for all the covariate-combinations found in the dataset.

   If `pop=FALSE` the function returns a list containing the following elements:

   `time` points at which the estimations were made

   `frag` number of subintervals

   `BGrad1.ls` a list each element of which contains a data.frame corresponding to $\frac{\partial F_1(t,\mathbf{x};\boldsymbol{\beta})}{\partial \beta_i}$ for a specific time point t

   `BGrad2.ls` a list each element of which contains a data.frame corresponding to $\frac{\partial F_2(t,\mathbf{x};\boldsymbol{\beta})}{\partial \beta_i}$ for a specific time point t

   `CPr1.df` and `CPr2.df` dataframes with all the event-specific probabilities for all individuals included in the dataset at each time point

   `CPr1Lo.df`, `CPr2Lo.df` dataframes which contain the lower limits of the event-specific probabilities for cause 1 and 2, respectively

   `CPr1Up.df`, `CPr2Up.df` dataframes which contain the upper limits of the event-specific probabilities for cause 1 and 2, respectively

   If `pop=TRUE` the values returned are:

   `time` time that the estimations were made

   `frag` number of subintervals

   `BGrad1.ls` a list each element of which is corresponding to $\left(\nabla F_1(t,\mathbf{x}_1;\boldsymbol{\beta})_{|\boldsymbol{\beta}=\widehat{\boldsymbol{\beta}}}, \cdots, \nabla F_1(t,\mathbf{x}_N;\boldsymbol{\beta})_{|\boldsymbol{\beta}=\widehat{\boldsymbol{\beta}}}\right)$ for a specific time point

   `BGrad2.ls` a list each element of which is corresponding to $\left(\nabla F_2(t,\mathbf{x}_1;\boldsymbol{\beta})_{|\boldsymbol{\beta}=\widehat{\boldsymbol{\beta}}}, \cdots, \nabla F_2(t,\mathbf{x}_N;\boldsymbol{\beta})_{|\boldsymbol{\beta}=\widehat{\boldsymbol{\beta}}}\right)$ for a specific time point

   `CPr.1` and `CPr.2` vectors of the population-level event-specific probabilities based on the whole dataset at each time point for cause 1 and 2, respectively

   `CPr1Lo`, `CPr2Lo` vectors of the lower limits of the event-specific probabilities for cause 1 and 2, respectively

   `CPr1Up`, `CPr2Up` vectors of the upper limits of the event-specific probabilities for cause 1 and 2, respectively

`CPr1.df` and `CPr2.df` dataframes with all the event-specific probabilities for all individuals included in the dataset at each time point

`NBGrad1.ls` and `NBGrad2.ls` lists corresponding to $\mathbf{w}^\top[\nabla F_j^{\text{Mat}}(t;\boldsymbol{\beta})]^\top_{|\boldsymbol{\beta}=\widehat{\boldsymbol{\beta}}}$ for $j = 1, 2$, respectively

`Var1` and `Var2` vectors with the estimated population-level variances of the cumulative probabilities at each time point for cause 1 and 2, respectively

`Var1b` and `Var2b` vectors with the estimated population-level variances of the transformed cumulative probabilities at each time point for cause 1 and 2, respectively

Both functions listed above are relying on other functions like the `cumIncidence.CS`, `BGrad_func`, etc, which are called internally by the aforementioned functions and not by the user.

Also, we have to note here that both functions (`csprob`, `predict.prob`) provide the individual estimates but the type of the resulted objects are different.

3.2 Estimation of the event-specific cumulative probabilities and their variances based on specific covariates

No matter if our goal is to provide individual or population predictions, we must predict first the covariate-specific point estimates and variances.

In the code below, we show how we can estimate the covariate-specific point estimates and confidence intervals and plot them using `ggplot2` for 3 random individuals.

```
fragm=1000
maxt=30

results <- csprob(mydata, Mod1, Mod2, maxt, fragm)

indivprob <- predict.prob(results, pop=FALSE)


# Choose a random sample of 3 individuals
ind <- sample(1:nrow(mydata),3)
randsam<- as.data.frame(
  cbind(
    'timesurv'=rep(indivprob$time[-1],3),
    'mean'  =c(indivprob$CPr2.df[,ind]),
    'upper' =c(indivprob$CPr2Up.df[,ind]),
    'lower' =c(indivprob$CPr2Lo.df[,ind]),
    'individual'=(rep(ind, each=fragm))
  ))
randsam$individual <- as.factor(randsam$individual)

#Plot
ggplot(data=randsam, aes(x=timesurv, y=mean)) +
  geom_line(aes(colour=individual)) +
  geom_ribbon(aes(ymax=upper, ymin=lower, fill=individual),
              alpha = 0.5)+
  theme_bw()+
  ggtitle("Covariate-specific predictions for Cause 2")+
  ylab("Cumulative Probability")
```

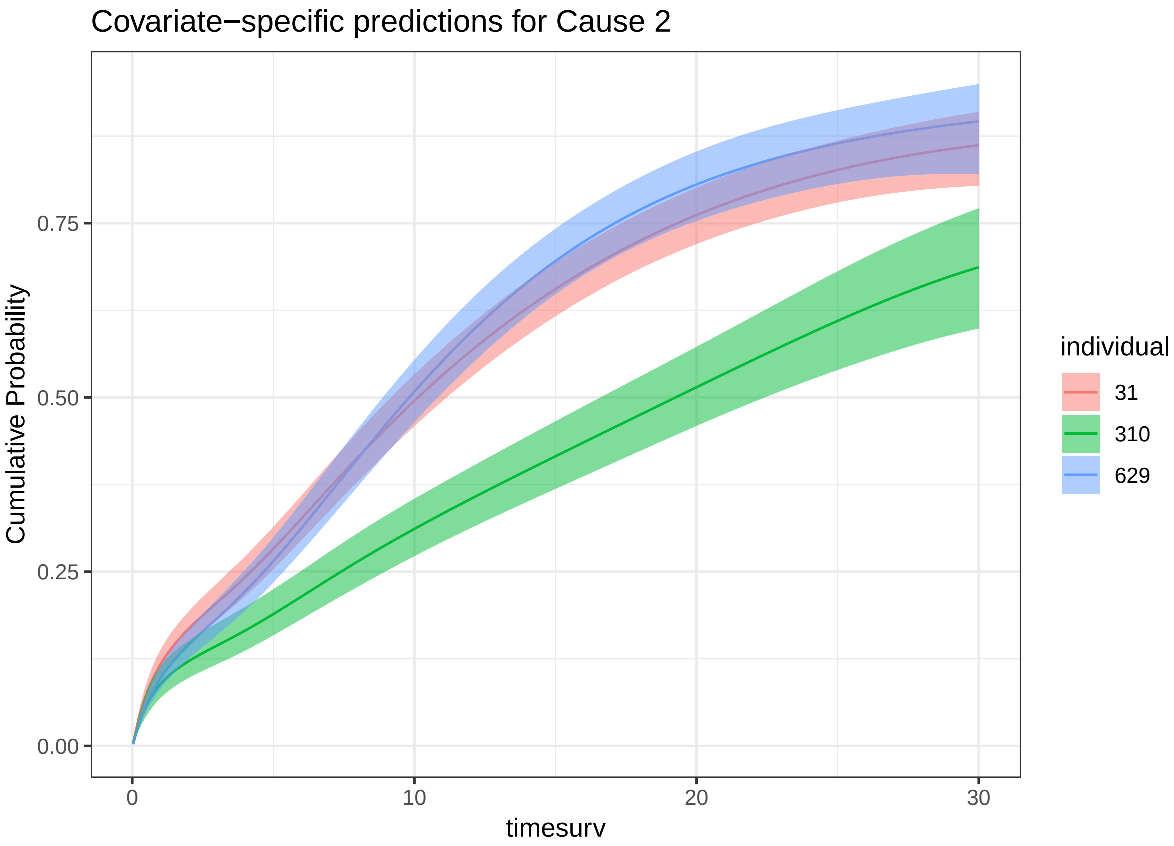

3.3 Estimation of the event-specific cumulative probabilities on the whole population with their variances

Finally, the population estimates are obtained as follows.

```
popprob<- predict.prob(results, pop=TRUE)

popdat <- as.data.frame(
  cbind('timesurv'=popprob$time,
        'mean'=c(popprob$CPr.1,popprob$CPr.2),
        'upper'=c(popprob$CPr1Lo,popprob$CPr2Lo),
        'lower'=c(popprob$CPr1Up,popprob$CPr2Up),
        'group'=(rep(1:2, each=fragm))
  ))

popdat$group <- as.factor(popdat$group)
levels(popdat$group) <- c("Cause 1","Cause 2")


ggplot(data=popdat, aes(x=timesurv, y=mean)) +
  geom_line(aes(colour=group)) +
  theme_bw()+
  geom_ribbon(aes(ymax=upper, ymin=lower, fill=group), alpha = 0.5)+
  ggtitle("Population estimates with their confidence intervals")+
  labs(y="Cumulative Probability")
```

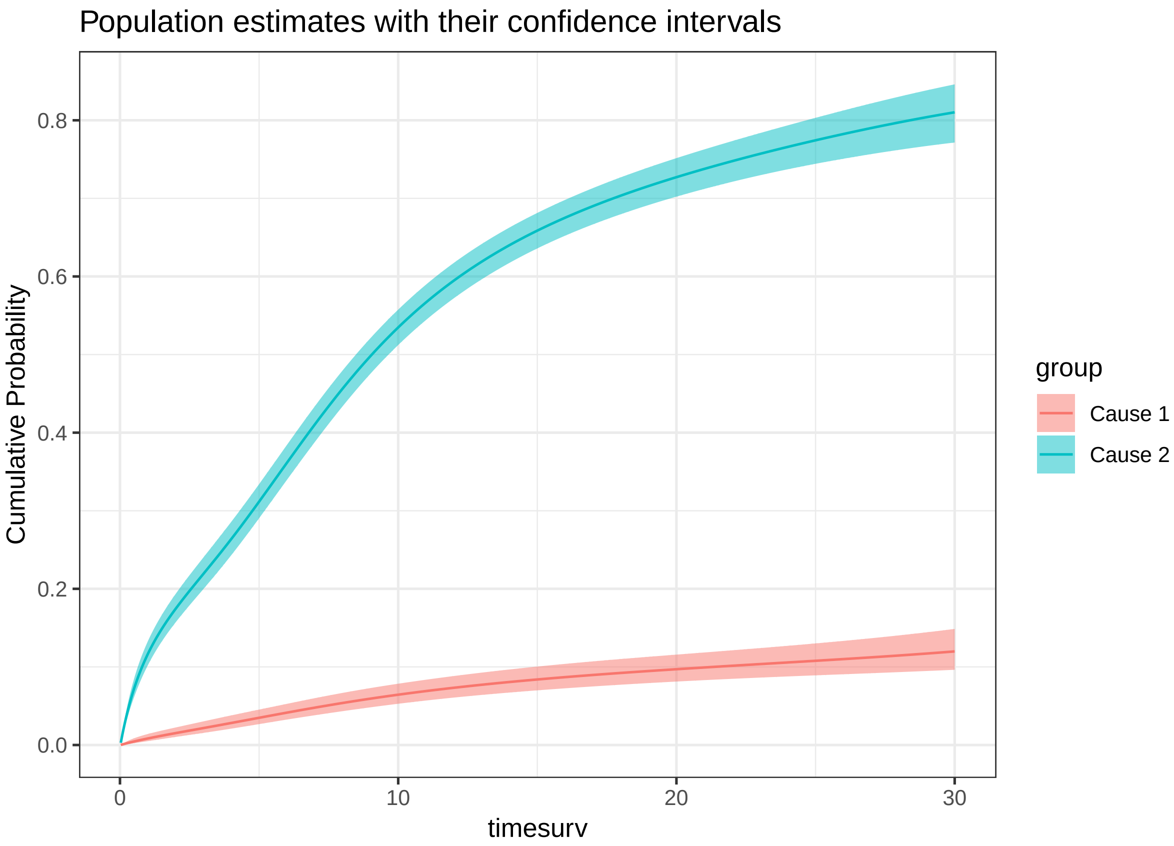

3.4 Dealing with a big dataset

Let us suppose that we have a quite big dataset and we want to decrease the execution time. We show here how to speed the calculations up by using the `doSNOW` package. The `doSNOW` package allows for parallel programming with function `foreach`. To make this run we need first to define how many *jobs* we want the computer to run at the same time and then run the loop through the whole dataset. A small example of the correspondence between `for` and `foreach` with `doSNOW` is shown below.

- `for` loop syntax

```
for (i in vector){
  <code>
    return(object)
}
```

- `foreach` loop syntax

```
foreach (i=vector, .combine='fun') %dopar% {
  <code*>
    return(object)
}
```

* For the `foreach` function, it is essential that the necessary packages be loaded via `library()` inside the loop. This is similar to having multiple new R windows. Alternatively, there is the `.packages` option within the loop specification.

An example of how we could replace `for` with `foreach` is shown below.

```
# clusters:  Number of slave nodes to create on the local machine
clusters<- 8
cl <- makeCluster(clusters, type = "SOCK")
registerDoSNOW(cl)


# Individual point estimates
csprobPar <- function(data_df, modA, modB, time.max, frag){
    foreach(y = 1:nrow(data_df),.combine = "rbind") %dopar% {
                  library("mexhaz")
                  library("Matrix")
                  source("CumIncid.R")

                  list(cumIncidence.CS(modA, modB,time.max,subdiv=frag,
                                  data.val=data_df[y,]))



            }
}

resultsPar <- csprobPar(mydata, Mod1, Mod2, maxt, fragm)

stopCluster(cl)

popprob<-predict.prob(resultsPar, pop=TRUE)
```

3.5 Adjusted probabilities and standardised risk differences

In section 2.2.3 we discussed about the adjusted probabilities and how we could quantify the effect of a variable on the cumulative probability scale. We show that here with the effect of interest being the effect of sex. To do so, we created 2 hypothetical populations, one where all patients were considered as women and another where all patients were considered as men, while keeping the other variables as observed. Practically, we did exactly the same as before when predicting the event-specific probabilities for the whole population with the only difference being the data used.

The function that provides the event-specific probabilities for both populations are the `csprob` and `predict.prob` applied to the data `mydataF` and `results_F` for women and `mydataM` and `results_M` for men. Using the predictions `ProbF` and `ProbM`, we can further estimate the differences and their 95% confidence intervals by using the function `csprobdif`.

Function `csprobdif` has the following syntax:

```
csprobdif(predprob1, predprob2)
```

where `predprob1` and `predprob2` are objects coming from the `predict.prob` function. The returned values from this function are

`time` vector of times of estimation

`ProbDif1`, `ProbDif2` estimated differences for cause 1 and 2, respectively

`ProbDif1Lo`, `ProbDif2Lo` the lower 95% confidence limits of the estimated differences for cause 1 and 2, respectively

ProbDif1Up, ProbDif2Up the upper 95% confidence limits of the estimated differences for cause 1 and 2, respectively

```
levels(mydata$sex)
```

[1] "F" "M"

```
for (i in 1:length(levels(mydata$sex))){
  mydataadj <- mydata
  mydataadj$sex<-as.factor(levels(mydata$sex)[i])
  assign(paste("mydata", levels(mydata$sex)[i],sep=""),mydataadj)

}


# csprob.predict
results_F<- csprob(data_df=mydataF, Mod1, Mod2, maxt, fragm)
results_M<- csprob(data_df=mydataM, Mod1, Mod2, maxt, fragm)

ProbF<-predict.prob(results_F, pop=TRUE)
ProbM<-predict.prob(results_M, pop=TRUE)

ProbDif<- csprobdif(ProbF, ProbM)


par(mfrow=c(2,2),oma = c(2, 1, 1, 1))

newdata<- data.frame(cbind("t"= ProbF$time,
                           "w2"=ProbF$CPr.2,"w2Lo"=ProbF$CPr2Lo,
                           "w2Up"=ProbF$CPr2Up,
                           "w1"=ProbF$CPr.1,"w1Lo"=ProbF$CPr1Lo,
                           "w1Up"=ProbF$CPr1Up,
                           "m2"=ProbM$CPr.2,"m2Lo"=ProbM$CPr2Lo,
                           "m2Up"=ProbM$CPr2Up,
                           "m1"=ProbM$CPr.1,"m1Lo"=ProbM$CPr1Lo,
                           "m1Up"=ProbM$CPr1Up))


dataPCM<- data.frame(cbind("Y"=c(newdata$w1,newdata$m1),
                            "L"=c(newdata$w1Lo,newdata$m1Lo),
                            "U"=c(newdata$w1Up,newdata$m1Up),
                            "X"=rep(newdata$t,2)))


dataD<- data.frame(cbind("Y"=c(newdata$w2,newdata$m2),
                            "L"=c(newdata$w2Lo,newdata$m2Lo),
                            "U"=c(newdata$w2Up,newdata$m2Up),
                            "X"=rep(newdata$t,2)))

dataPCM$gender <- dataD$gender <- as.factor(rep(c(2,1),each=fragm))
levels(dataPCM$gender) <- levels(dataD$gender)<-  c("men", "women")
```

```
plotPCM<- ggplot(data=dataPCM, aes(x=X, y=Y)) +
  geom_line(aes(linetype=gender, color=gender)) +
  geom_ribbon(aes(ymax=U, ymin=L, fill=gender), alpha = 0.5)+
  theme_bw()+
  labs(x="Time (in years)")+
  theme(legend.position="top",plot.title = element_text(hjust = 0.5))+
  ggtitle("PCM")+
  ylab("Adjusted cumulative probability")

plotD<- ggplot(data=dataD, aes(x=X, y=Y)) +
  geom_line(aes(linetype=gender)) +
  theme_bw()+
  theme(legend.position="top",plot.title = element_text(hjust = 0.5))+
  geom_ribbon(aes(ymax=U, ymin=L, fill=gender), alpha = 0.5)+
  ggtitle("Death w/o malignancy")+
  labs(x="Time (in years)")+
  ylab("Adjusted cumulative probability")




dataDifPCM  <- data.frame(cbind("Y"=ProbDif$ProbDif1,
                                "L"=ProbDif$ProbDif1Lo,
                                "U"=ProbDif$ProbDif1Up,
                                "X"=ProbDif$time))


dataDifD  <-  data.frame(cbind("Y"=ProbDif$ProbDif2,
                               "L"=ProbDif$ProbDif2Lo,
                               "U"=ProbDif$ProbDif2Up,
                               "X"=ProbDif$time))


plotDifPCM<-ggplot(data=dataDifPCM, aes(x=X, y=Y)) +
                    geom_line() +
                    theme_bw()+
                    geom_hline(yintercept=0, linetype="dashed")+
                    geom_ribbon(aes(ymax=U, ymin=L), alpha = 0.3)+
                    ggtitle("")+
                    labs(x="Time (in years)")+
                    ylab("Standardised risk difference \n (women-men)")

plotDifD<-ggplot(data=dataDifD, aes(x=X, y=Y)) +
                    geom_line() +
                    theme_bw()+
                    geom_hline(yintercept=0, linetype="dashed")+
                    geom_ribbon(aes(ymax=U, ymin=L), alpha = 0.3)+
                    ggtitle("")+
                    labs(x="Time (in years)")+
                    ylab("Standardised risk difference \n (women-men)")
```

Figure 1: Adjusted cumulative probabilities of progressing to plasma cell malignancy, PCM, (left top panel) and to death (right top panel) for men and women, and average effect of sex for PCM (left bottom panel) and death from other cause (right bottom panel).

```r
grid.arrange(plotPCM, plotD,
                    plotDifPCM, plotDifD ,ncol=2)
```

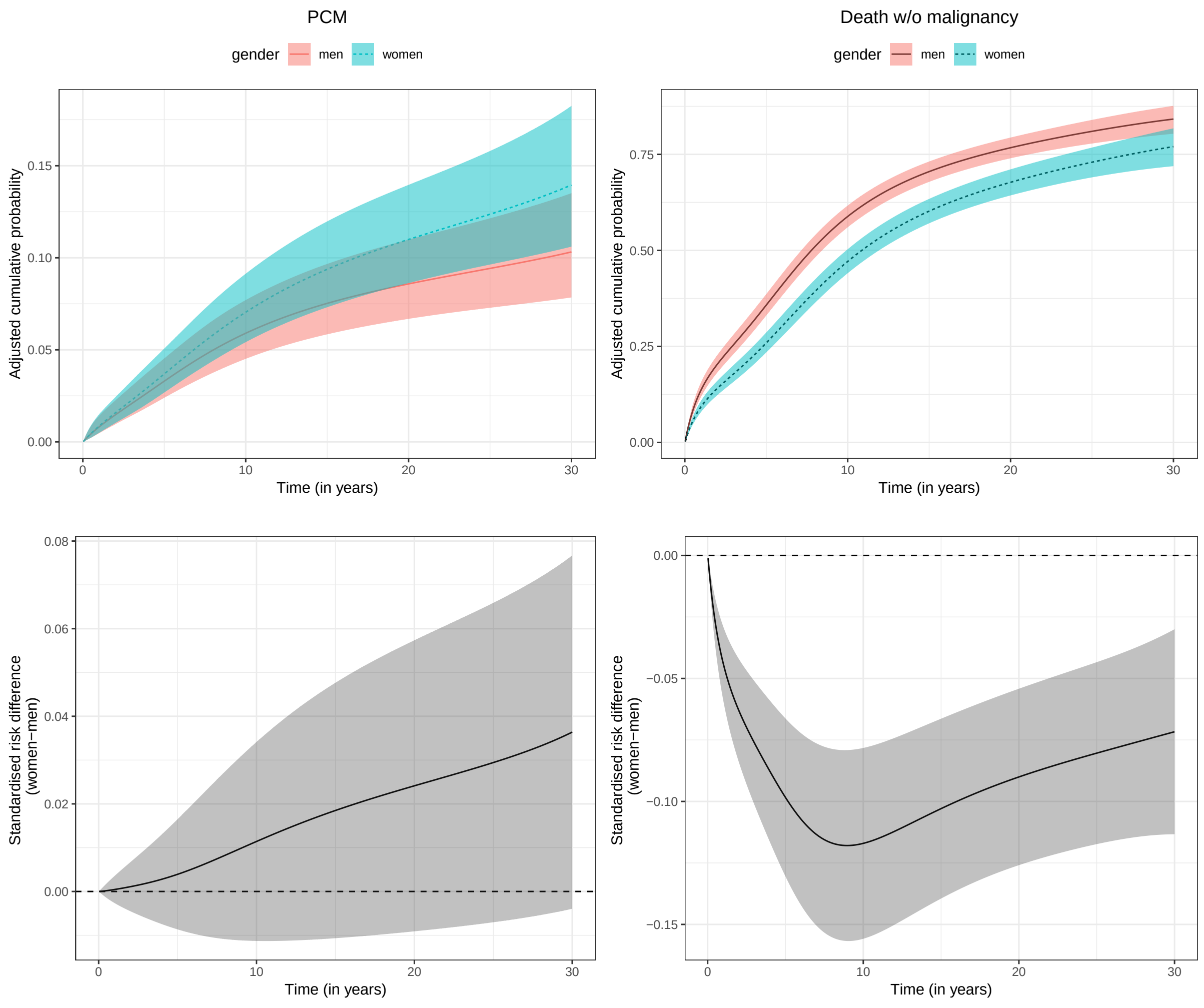

CumIncid.R

Please run these before starting any calculations.

```
# Cumulative incidence using two cause-specific models
cumIncidence.CS <- function(model1,model2,time.max,subdiv,
                            data.val=data.frame(.NotUsed=NA),alpha=0.05){
  time.pts <- seq(0,time.max,le=(subdiv+1))
  CstMult <- time.max/(2*subdiv)
  CstCI <- qnorm(1-alpha/2)

  # Or if you want to take into account the size of the population
  # CstCI <- qt(1-alpha/2,df=model£n.obs)

  Pred1 <- predict(model1,time.pts,data.val, include.gradient=T)
  Pred2 <- predict(model2,time.pts,data.val, include.gradient=T)

  ISurv1 <- Pred1$results$hazard*Pred1$results$surv*Pred2$results$surv
  CPr.1 <- cumsum(ISurv1+c(0,ISurv1[-subdiv]))*(time.max/(2*subdiv))

  ISurv2 <- Pred2$results$hazard*Pred1$results$surv*Pred2$results$surv
  CPr.2 <- cumsum(ISurv2+c(0,ISurv2[-subdiv]))*(time.max/(2*subdiv))

  ISurvT <- (Pred1$results$hazard+Pred2$results$hazard)*Pred1$results$surv*
                Pred2$results$surv
  CPrT<- cumsum(ISurvT+c(0,ISurvT[-subdiv]))*(time.max/(2*subdiv))
  SurvT <- 1-CPrT

  # Confidence intervals
  which.td1<- rownames(Pred1$vcov)[-c(1,which(rownames(Pred1$vcov)
                                          %in% model1$names.ph))]
  which.ntd1 <- c(rownames(Pred1$vcov)[-c(which(rownames(Pred1$vcov)
                                            %in% model1$names.ph))][1],
                rownames(Pred1$vcov)[which(rownames(Pred1$vcov) %in%
                                           model1$names.ph)])

  which.td2<- rownames(Pred2$vcov)[-c(1,which(rownames(Pred2$vcov) %in%
                                            model2$names.ph))]
  which.ntd2 <- c(rownames(Pred2$vcov)[-c(which(rownames(Pred2$vcov) %in%
                                              model2$names.ph))][1],
                rownames(Pred2$vcov)[which(rownames(Pred2$vcov) %in%
                                           model2$names.ph)])

  Vcov1 <- model1$vcov[c(which.ntd1,which.td1),c(which.ntd1,which.td1)]
  Vcov2 <- model2$vcov[c(which.ntd2,which.td2),c(which.ntd2,which.td2)]
  CovMat <- as.matrix(bdiag(Vcov1,Vcov2))
  AGrad11 <- (Pred1$grad.loghaz + Pred1$grad.logcum*(log(Pred1$results$surv)))
                  [,c(which.ntd1,which.td1)]
  AGrad12 <- (Pred2$grad.logcum*(log(Pred2$results$surv)))
                  [,c(which.ntd2,which.td2)]
  AGrad21 <- (Pred1$grad.logcum*(log(Pred1$results$surv)))
                  [,c(which.ntd1,which.td1)]
```

```
  AGrad22 <- (Pred2$grad.loghaz + Pred2$grad.logcum*(log(Pred2$results$surv)))
                  [,c(which.ntd2,which.td2)]

  AGrad1 <- cbind(AGrad11,AGrad12)
  AGrad2 <- cbind(AGrad21,AGrad22)
  AGradT <- cbind(AGrad21,AGrad12)
  Temp1 <- ISurv1*AGrad1
  Denom1 <- (1-CPr.1)*log(1-CPr.1)
  BGrad1 <- apply(Temp1,2,function(x) cumsum(x+c(0,x[-subdiv]))*
                  (time.max/(2*subdiv)))

  TMatVar1 <- CovMat%*%t(BGrad1)
  Var1 <- sapply(1:subdiv,function(i) BGrad1[i,]%*%TMatVar1[,i])
  Var1b <- Var1/((1-CPr.1)*log(1-CPr.1))^2

  Temp2 <- ISurv2*AGrad2
  Denom2 <- (1-CPr.2)*log(1-CPr.2)^2
  BGrad2 <- apply(Temp2,2,function(x) cumsum(x+c(0,x[-subdiv]))*
                  (time.max/(2*subdiv)))

  TMatVar2 <- CovMat%*%t(BGrad2)
  Var2 <- sapply(1:subdiv,function(i) BGrad2[i,]%*%TMatVar2[,i])
  Var2b <- Var2/((1-CPr.2)*log(1-CPr.2))^2

  BGradT <- AGradT/log(SurvT)
  TMatVarT <- CovMat%*%t(BGradT)
  VarT <- sapply(1:subdiv,function(i) BGradT[i,]%*%TMatVarT[,i])
  Transf <- function(x,vx,m){
    log(-log(x))+m*CstCI*sqrt(vx)
  }
  InvTransf <- function(x){
    exp(-exp(x))
  }
  Cr1Lo <- 1-InvTransf(Transf(1-CPr.1,Var1b,-1))
  Cr1Up <- 1-InvTransf(Transf(1-CPr.1,Var1b,1))
  Cr2Lo <- 1-InvTransf(Transf(1-CPr.2,Var2b,-1))
  Cr2Up <- 1-InvTransf(Transf(1-CPr.2,Var2b,1))
  CrTLo <- 1-InvTransf(Transf(SurvT,VarT,-1))
  CrTUp <- 1-InvTransf(Transf(SurvT,VarT,1))

  return(list("time"= time.pts,
              "frag"=subdiv,
              "CPr.1"=CPr.1,"CPr.2"=CPr.2,"CovMat"=CovMat,
              "BGrad1"=BGrad1,"BGrad2"=BGrad2,"CPr1Lo"= Cr1Lo,
              "CPr1Up"=Cr1Up,"CPr2Lo"= Cr2Lo,"CPr2Up"=Cr2Up,
              "Var1"=Var1, "Var2"=Var2,"Var1b"=Var1b,"Var2b"=Var2b))
}
#Functions for prediction
csprob<-function(data_df, modA, modB, time.max, frag){
  results_list<- list()
  for (y in 1:nrow(data_df)){
```

```
    results_list[[y]] <- cumIncidence.CS(modA, modB,time.max,
                                          subdiv=frag,
                                          data.val=data_df[y,])
  }
  return(results_list)
}


BGrad_func <- function(BGrad.ls,frag,N, p_dim){
  BGrad.3da <- array(unlist(BGrad.ls),dim = c(frag,p_dim,N))
  BGrad.3d  <- aperm(BGrad.3da,dim=c(3,1,2))
  NBGrad.ls <- alply(BGrad.3d,3)
  return(NBGrad.ls)
}

Var_func <- function (x,w,NBGrad, CovMat){
  BGrad<- w%*%NBGrad[[x]]
  TMatVar <- CovMat%*%t(BGrad)
  res<-  BGrad%*%TMatVar
  return(res)
}

Transf <- function(x,vx,m){log(-log(x))+m*qnorm(0.975)*sqrt(vx)}
InvTransf <- function(x){exp(-exp(x))}

predict.prob <- function(csprobObj, pop){

  CovMat <- csprobObj[[1]]$CovMat
  p_dim <- dim(csprobObj[[1]]$CovMat)[1]
  N <- length(csprobObj)
  frag <- csprobObj[[1]]$frag

  if (pop==FALSE){
    BGrad1.ls <- lapply(1:N, function(x) csprobObj[[x]]$BGrad1)
    BGrad2.ls <- lapply(1:N, function(x) csprobObj[[x]]$BGrad2)
    CPr1.df <- sapply(1:N, function(x) csprobObj[[x]]$CPr.1)
    CPr2.df <- sapply(1:N, function(x) csprobObj[[x]]$CPr.2)
    CPr1Lo.df <- sapply(1:N, function(x) csprobObj[[x]]$CPr1Lo)
    CPr1Up.df <- sapply(1:N, function(x) csprobObj[[x]]$CPr1Up)
    CPr2Lo.df <- sapply(1:N, function(x) csprobObj[[x]]$CPr2Lo)
    CPr2Up.df <- sapply(1:N, function(x) csprobObj[[x]]$CPr2Up)

    return(list("time"=csprobObj[[1]]$time,"frag"=frag,
                "BGrad1.ls"=BGrad1.ls, "BGrad2.ls"=BGrad2.ls,
                "CPr1Lo.df"=CPr1Lo.df,"CPr1Up.df"=CPr1Up.df,
                "CPr2Lo.df"=CPr2Lo.df,"CPr2Up.df"=CPr2Up.df,
                "CPr1.df"=CPr1.df,"CPr2.df"=CPr2.df))
  }
  if (pop==TRUE){
    w <- matrix(rep(1/N,N), nrow =1)
```

```
    BGrad1.ls<- lapply(1:N, function(x) csprobObj[[x]]$BGrad1)
    BGrad2.ls<- lapply(1:N, function(x) csprobObj[[x]]$BGrad2)
    CPr1.df <- sapply(1:N, function(x) csprobObj[[x]]$CPr.1)
    CPr2.df <- sapply(1:N, function(x) csprobObj[[x]]$CPr.2)

    CPr.1 <- w%*%t(CPr1.df)
    CPr.2 <- w%*%t(CPr2.df)

    NBGrad1.ls <- BGrad_func(BGrad1.ls,frag,N,p_dim)
    NBGrad2.ls <- BGrad_func(BGrad2.ls,frag,N,p_dim)

    Var1 <- data.frame(sapply(1:frag, function(x)
                            Var_func(x,w,NBGrad1.ls, CovMat) ))
    Var1b <- Var1/((1-CPr.1)*log(1-CPr.1))^2
    CPr1Lo <- 1-InvTransf(Transf(1-CPr.1,Var1b,-1))
    CPr1Up <- 1-InvTransf(Transf(1-CPr.1,Var1b,1))

    Var2 <- data.frame(sapply(1:frag, function(x)
                            Var_func(x,w,NBGrad2.ls, CovMat) ))
    Var2b <- Var2/((1-CPr.2)*log(1-CPr.2))^2
    CPr2Lo <- 1-InvTransf(Transf(1-CPr.2,Var2b,-1))
    CPr2Up <- 1-InvTransf(Transf(1-CPr.2,Var2b,1))
    return(list("time"=csprobObj[[1]]$time[-1],"frag"=frag,
                "BGrad1.ls"=BGrad1.ls, "BGrad2.ls"=BGrad2.ls,
                "CPr.1"=CPr.1[1,],"CPr.2"=CPr.2[1,],"NBGrad1.ls"=NBGrad1.ls,
                "NBGrad2.ls"=NBGrad2.ls,"Var1"=Var1,"Var2"=Var2,
                "Var1b"=Var1b,"Var2b"=Var2b,
                "CPr1Lo"=CPr1Lo[,1],"CPr1Up"=CPr1Up[,1],
                "CPr2Lo"=CPr2Lo[,1],"CPr2Up"=CPr2Up[,1],
                "CPr1.df"=CPr1.df,"CPr2.df"=CPr2.df,"CovMat"=CovMat))
  }
}




# Calculate differences between populations
csprobdif<- function(predprob1, predprob2){

  N <- ncol(predprob1$CPr1.df)
  w<- matrix(rep(1/N,N), nrow =1)
  frag <- predprob1$frag
  CovMat <- predprob1$CovMat

  NBGrad1.ls <- lapply(1:frag, function (x) predprob1$NBGrad1.ls[[x]]-
                       predprob2$NBGrad1.ls[[x]])
  NBGrad2.ls <- lapply(1:frag, function (x) predprob1$NBGrad2.ls[[x]]-
                       predprob2$NBGrad2.ls[[x]])

  CP1.dif_df <- predprob1$CPr1.df-predprob2$CPr1.df
  CP2.dif_df  <- predprob1$CPr2.df-predprob2$CPr2.df
```

```
  CP1.dif <-apply(CP1.dif_df,1,mean)
  CP2.dif<-apply(CP2.dif_df,1,mean)

  ci_form <- function(x,var,m){
    x+m*qnorm(0.975)*sqrt(var)
  }

  Var1 <-data.frame(sapply(1:frag, function(x)
                              Var_func(x,w,NBGrad1.ls,CovMat)))
  Var2 <-data.frame(sapply(1:frag, function(x)
                              Var_func(x,w,NBGrad2.ls,CovMat)))

  CPr1.difLo <- ci_form(CP1.dif,Var1,-1)[,1]
  CPr1.difUp <- ci_form(CP1.dif,Var1,+1)[,1]
  CPr2.difLo <- ci_form(CP2.dif,Var2,-1)[,1]
  CPr2.difUp <- ci_form(CP2.dif,Var2,+1)[,1]
  return(list("time"= predprob1$time,
              "ProbDif1"=CP1.dif, "ProbDif2"=CP2.dif,
              "ProbDif1Lo"=CPr1.difLo,"ProbDif1Up"=CPr1.difUp,
              "ProbDif2Lo"=CPr2.difLo,"ProbDif2Up"=CPr2.difUp))

}
```
